# Haemoptysis in chronic pulmonary vascular disease: a narrative review

**DOI:** 10.1183/16000617.0055-2026

**Published:** 2026-10-21

**Authors:** Jan C. Kamp, Brandon Budhram, Mitja Jevnikar, Karen M. Olsson, Matti J. Peperhove, Lavinia Neubert, Marius M. Hoeper, Marc Humbert

**Affiliations:** 1 Department of Respiratory Medicine and Infectious Diseases, Hannover Medical School, Hannover, Germany; 2 Biomedical Research in Endstage and Obstructive Lung Disease Hannover (BREATH), German Center for Lung Research (DZL), Hannover, Germany; 3 Division of Respirology, Department of Medicine, University of Calgary, Calgary, AB, Canada; 4 Université Paris-Saclay, Inserm UMR_S 1358 (HPPIT), Department of Respiratory and Intensive Care Medicine, Hôpital Bicêtre (AP-HP), FHU André Cournand, ERN-LUNG, Le Kremlin-Bicêtre, France; 5 Institute for Diagnostic and Interventional Radiology, Hannover Medical School, Hannover, Germany; 6 Institute of Pathology, Hannover Medical School, Hannover, Germany; 7 These authors contributed equally as senior authors to the manuscript

## Abstract

Haemoptysis, the expectoration of blood from the lower airways, is a complication of several pulmonary vascular diseases (PVDs). The pathophysiology of haemoptysis in chronic PVDs is complex and varies depending on the underlying condition. Computed tomography pulmonary angiography remains the gold standard for localising the bleeding source and guiding interventional therapies. Management of haemoptysis in patients with chronic PVDs can range from oxygen supplementation to angiography-guided bronchial artery embolisation, depending on bleeding severity, anatomical features and specific features of the associated disease. Regardless of the management strategy, long-term follow-up is essential in these patients owing to the risk of recurrent bleeding. The aim of this review is to comprehensively explore the characteristics of haemoptysis in selected chronic PVDs, summarise current knowledge of the pathophysiology, and provide guidance on contemporary diagnostic and treatment strategies.

## Shareable abstract

**Haemoptysis is a rare but potentially life-threatening complication of chronic pulmonary vascular diseases. This review summarises the current knowledge of its pathophysiology and provides guidance on contemporary diagnostic and treatment strategies.** https://bit.ly/4bEObtV

## Introduction

Haemoptysis is a clinical symptom with a wide spectrum of severities that can affect patients with pulmonary vascular diseases (PVDs). Depending on the underlying condition, the pathophysiology, clinical presentation and management of haemoptysis may vary. To understand the complex mechanisms and disease-specific hallmarks of haemoptysis and to summarise current diagnostic strategies and treatment principles, we performed an extensive review of the literature. We focused on the manifold entities of pulmonary hypertension (PH) and other chronic PVDs with an increased risk of haemoptysis, including hereditary haemorrhagic telangiectasia, Behcet's disease and Hughes–Stovin syndrome (HSS). Haemoptysis associated with acute pulmonary embolism, pulmonary oedema due to left heart disease, lung cancer, pulmonary infections and pulmonary vasculitides were not the subject of this review.

### Overview of pathophysiology, diagnosis and management

In patients with chronic PVDs, haemoptysis arises from a complex interplay between general risk factors and disease-specific mechanisms (figure 1). Although haemoptysis can originate from pulmonary arteries, pulmonary capillaries, systemic-to-pulmonary collaterals or even pulmonary veins, the bronchial arteries are most frequently implicated owing to their high-pressure flow and tendency for hypertrophy in chronic hypoxic or inflammatory states [1, 2]. The risk of haemoptysis correlates with increased pulmonary arterial pressure (PAP) [3, 4], infiltrative and erosive vascular inflammation [5, 6] and structural alterations such as pulmonary arterial pseudoaneurysms. Haemoptysis is encountered in various vasculopathies associated with pathogenic genetic variants causing obliterative pulmonary arterial/venous remodelling or progressive formation of arteriovenous malformations (AVMs) [7–13]. Additional risk factors include thrombocytopenia, disease-related impairment of haemostatic capacity and the use of therapeutic anticoagulation and/or antiplatelet agents [14–20].

**FIGURE 1:**
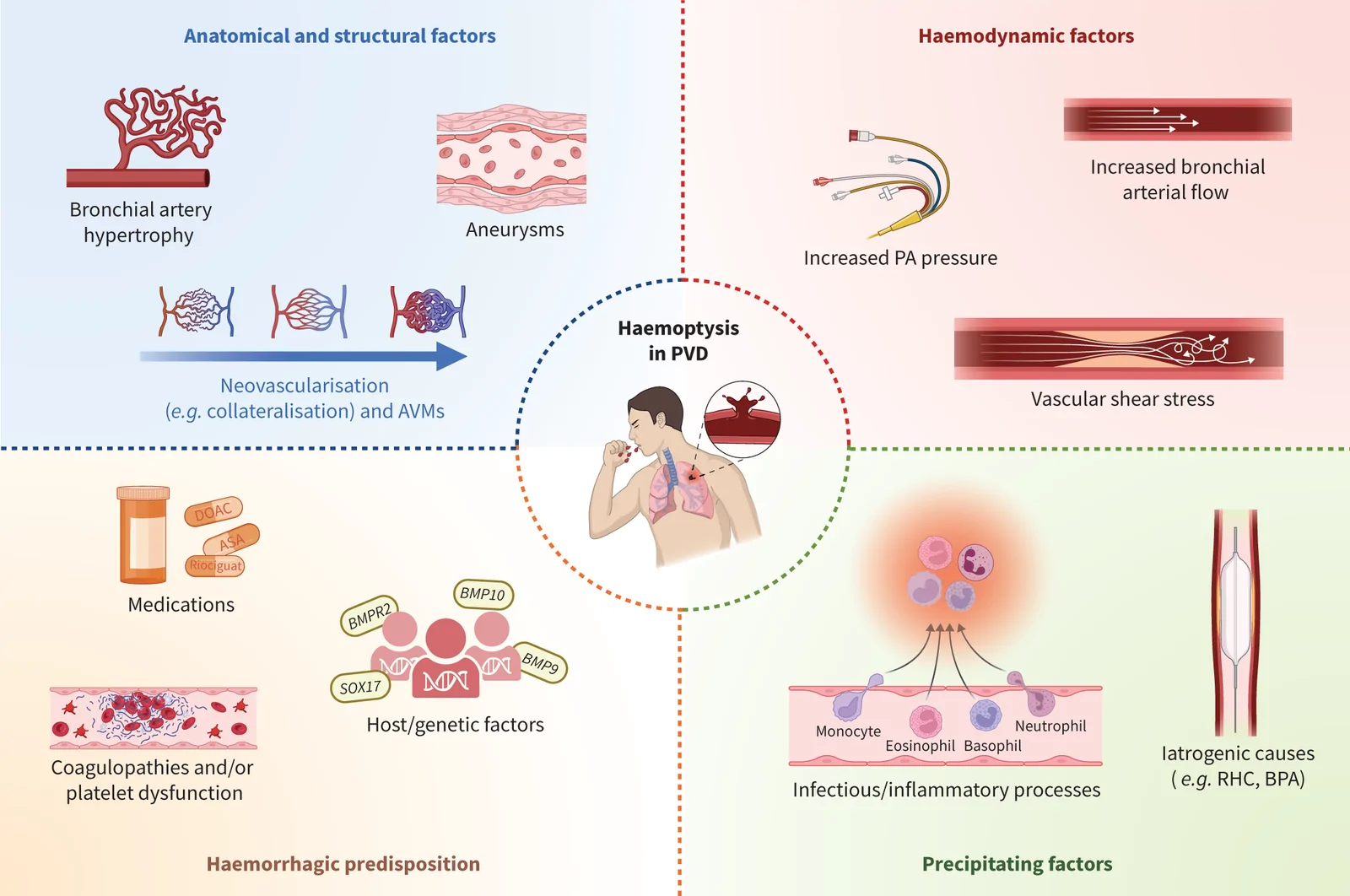
Pathophysiology of haemoptysis in chronic pulmonary vascular diseases (PVDs). ASA: acetylsalicylic acid; AVM: arteriovenous malformation; BPA: balloon pulmonary angiography; DOAC: direct oral anticoagulant; PA: pulmonary artery; RHC: right heart catheterisation.

The evaluation of haemoptysis events in chronic PVDs is influenced by the underlying disease, because specific anatomical features, such as collateral vessels and bronchial artery hypertrophy, elevate the risk of bleeding complications. The cornerstone of diagnosing the source of bleeding in patients with haemoptysis is computed tomography angiography (CTA) [21, 22]. CTA often enables identification of bleeding sources while providing detailed anatomical visualisation, which is critical for guiding advanced diagnostic and therapeutic techniques [23–25]. Monophasic CTA (*i.e.* single-phase injection of contrast medium in a constant flow) allows identification of hypertrophic bronchial arteries and non-bronchial systemic arterial supply to the lung, pulmonary artery branch pseudoaneurysms, and AVMs. Extravasation is rarely seen in clinical practice. In case of vascular abnormalities, biphasic CTA (*i.e.* injection of contrast medium in two consecutive phases with differing inflow velocities) can be considered to improve diagnostic accuracy. Early CTA is critical, because the capacity to visualise the bleeding source diminishes as blood accumulation increases within the pulmonary parenchyma.

Among diagnostic modalities, CTA often surpasses bronchoscopy in terms of diagnostic utility for chronic PVDs. However, bronchoscopy still serves a complementary role, particularly in cases of proximal bleeding, when the source of haemorrhage remains unclear following noninvasive testing, when the degree of extravasation does not allow a detection *via* CTA, or when occult alveolar haemorrhage is suspected and bronchoalveolar lavage is indicated [26, 27]. In addition to imaging- or instrument-based techniques, coagulation status and total blood count should always be assessed to rule out haemorrhagic diathesis due to impaired coagulation or thrombocytopenia. An overview of the diagnostic strategy is given in figure 2.

**FIGURE 2:**
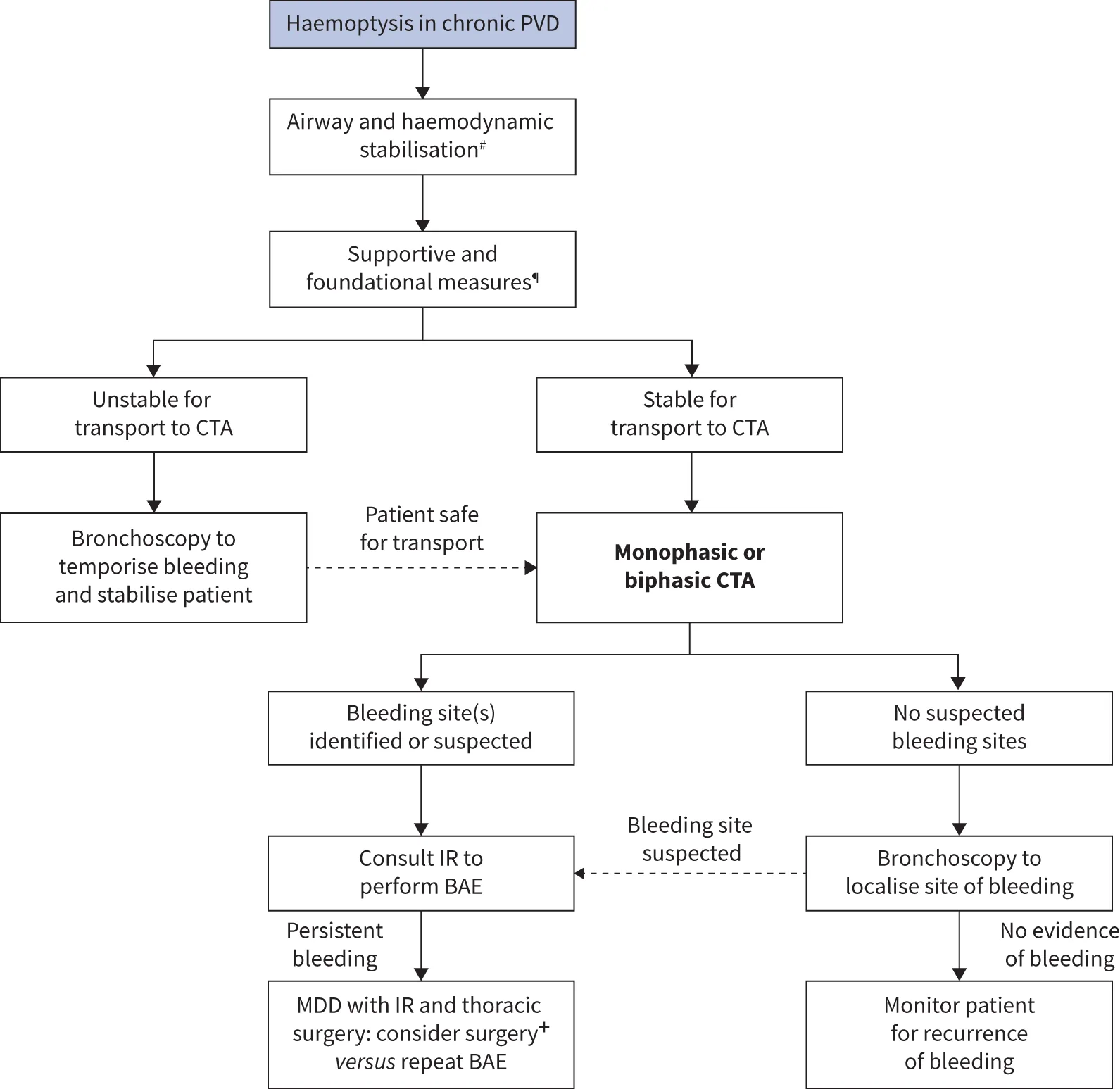
Diagnostic algorithm. BAE: bronchial artery embolisation; CTA: computed tomography angiography; IR: interventional radiology; MDD: multidisciplinary discussion; PVD: pulmonary vascular disease. ^#^: intubation should be avoided in patients with moderate-to-severe pulmonary hypertension when possible; ^¶^: supportive and foundational measures may include oxygenation, positioning (bleeding side down), suctioning, correction of coagulopathies, transfusion of blood products, *etc*.; ^+^: surgery should be avoided in patients with moderate-to-severe pulmonary hypertension when possible.

Treatment of haemoptysis in chronic PVDs is guided by patient stability, bleeding severity and the underlying disease. In stable patients, oxygen supplementation, volume resuscitation and correction of coagulopathies is recommended. In unstable patients, airway protection and cardiopulmonary stabilisation should follow established ABC (Airway, Breathing, Circulation) protocols, which may include endotracheal intubation, mechanical ventilation and haemodynamic support [28]. For persistent or severe bleeding, interventional procedures such as angiography and bronchial artery embolisation (BAE) are indicated following prompt CTA.

Bronchoscopy remains a valuable therapeutic option in select cases, particularly in the setting of life-threatening haemoptysis, allowing for bronchial blocker placement, selective intubation or topical application of vasoactive agents (*e.g.* epinephrine) [29–32]. Rigid bronchoscopy permits the use of larger instruments in central airways, while flexible bronchoscopy is better suited for peripheral visualisation and treatment [33, 34].

## Methods/search strategy

The literature search was conducted in MEDLINE using the terms “haemoptysis”, “pulmonary haemorrhage”, “pulmonary bleeding”, “pulmonary vascular disease”, “pulmonary arterial hypertension”, “PAH”, “chronic thromboembolic pulmonary hypertension”, “CTEPH”, “Takayasu arteritis”, “hereditary haemorrhagic telangiectasia”, “Hughes­­–Stovin syndrome” and “Behcet's disease”. The initial search included research articles published from 01 January 1990 up to 10 August 2025. Studies published after this date that are important to the field were manually added. Sources of unpublished studies and grey literature were not considered. No restrictions were applied regarding study design, end-points or language.

## Haemoptysis in pulmonary arterial hypertension

Pulmonary arterial hypertension (PAH) encompasses several forms of precapillary PH, including idiopathic and heritable forms, PAH associated with congenital heart disease (CHD-PAH) or connective tissue disease, and pulmonary veno-occlusive disease (PVOD). PAH is characterised by a significant increase in mean PAP and pulmonary vascular resistance (PVR) leading to right ventricular dysfunction. Haemoptysis can occur during the disease course, especially in patients with idiopathic and heritable PAH, though data on incidence, event severity and prognostic relevance from large cohort studies are lacking.

Although haemoptysis can occur in all forms of PAH, certain subgroups are particularly predisposed. CHD-PAH constitutes a heterogenous population, primarily consisting of patients with atrial or ventricular septal defects that cause left-to-right shunting of blood, resulting in chronic right ventricular overload and, eventually, PAH. In case of large septal defects, shunt reversal leading to severe hypoxaemia can occur once the right ventricular pressure exceeds the left, known as Eisenmenger syndrome. However, CHD-PAH can also occur in patients with small, subclinical septal defects, including those that persist or reoccur after surgical/interventional correction [35–37]. Patients with CHD-PAH are at risk for severe haemoptysis as a potentially life-threatening complication, with an overall incidence of 3.1–5.5% [38, 39] and an increased incidence of 6–49% in patients with Eisenmenger syndrome [40–44].

PVOD is a rare form of PAH, accounting for 5–10% of cases initially considered to be idiopathic disease [45]. It is driven by a progressive obstruction of small pulmonary veins leading to elevation in PVR and right ventricular failure [46, 47]. Heritable PVOD is considered a monogenetic autosomal recessive disease associated with biallelic mutations in the eukaryotic translation initiation factor-2α kinase 4 (*EIF2AK4*) gene [48], though several diseases and drug exposures can provoke PVOD. Other genetic mutations can predispose to haemoptysis in heritable PAH, including pathogenic variants of bone morphogenetic protein receptor 2 (*BMPR2*) and SRY-box transcription factor 17 (*SOX17*) [7, 8].

In PAH, there is an obliterative remodelling of the precapillary pulmonary vascular bed leading to increased PAP and PVR [3, 4]. Consequently, injury to the central/mediastinal or peripheral pulmonary arteries can occur either spontaneously or during right heart catheterisation [49–52], particularly in case of pulmonary artery pseudoaneurysms [2, 53, 54]. However, the vast majority of haemoptysis events in PAH originate from the bronchial circulation [1, 2], particularly in progressive PAH, given that increasing haemodynamic severity correlates with an increasing number of hypertrophied bronchial arteries [55, 56].

Certain patients with heritable PAH are at increased risk for haemoptysis, including those with pathogenic variants of *BMPR2* (substantial haemoptysis >50 mL) and *SOX17* (recurrent haemoptysis) [7, 8]. The mechanisms by which these genetic alterations increase the risk of haemoptysis remain unclear, though variants of the *BMPR2* gene are associated with an increased disease severity [57]. Similarly, variants in the *SOX17* gene can cause a severe PAH phenotype, frequently associated with congenital atrial or ventricular septal defects and pulmonary vascular malformations.

Furthermore, approximately 15% of patients with variants in the *GDF2* and *BMP10* genes encoding for BMP9/BMP10 have been reported to present with haemoptysis at diagnosis [58]. The high prevalence of haemoptysis at diagnosis in patients with *GFD2*/*BMP10*-associated heritable PAH points to a causative role of these genetic variants. There might be a cross-link to the novel group of activin signalling inhibitors with sotatercept as the first-in-class agent. Interestingly, telangiectasias and mild bleeding complications such as epistaxis are frequent side-effects of sotatercept [59, 60]. Furthermore, a recent study revealed decreased levels of the circulating vascular quiescence factors BMP9 and BMP10 24 weeks after sotatercept initiation [61]. These findings raise the possibility that genetically determined BMP9/BMP10 dysfunction may play a role in the pathophysiology of haemoptysis.

In PAH, there is an increased density of inflammatory cells in proximity to pulmonary vascular lesions that drives pulmonary vascular remodelling [5, 6] and an expansion of the pulmonary microvascular plexus through increased liberation of pro-angiogenic factors such as vascular endothelial growth factor (VEGF) and angiopoietin-1 [62]. This microenvironment stimulates the development of collateral vessels that usually originate from systemic arteries [63] and are often thin-walled and therefore prone to rupture [64]. Perivascular inflammation is also present around systemic vessels in PAH and might contribute to the development of haemoptysis by eroding the barrier between bronchial arteries and adjacent bronchi [23, 65].

The role of PAH therapies in the context of haemoptysis remains unclear. Prostacyclin analogues, as an example, are known to cause platelet dysfunction. In addition, inhaled PAH therapies have been suspected to provoke haemoptysis. However, a systematic review of treatment-emergent haemoptysis events in clinical studies enrolling patients with PAH showed no indication that PAH treatments are associated with an increased risk of pulmonary bleeding events (summarised in supplementary tables S1 and S2). The overall number of reported haemoptysis events in the 49 studies comprising the systematic review was low (48 events in 12 054 patients), though additional events occurred in long-term extension studies. The majority of haemoptysis events were reported in two studies: 1) the PATENT-2 open-label extension study, in which 25 events occurred in patients on open-label riociguat [66]; and 2) the AIR trial, comparing inhaled iloprost in PAH and placebo, in which five and two events occurred in the treatment and placebo group, respectively [67]. The phase 2 PULSAR and phase 3 STELLAR, ZENITH and HYPERION studies had a specific focus on sotatercept and mild bleeding complications such as epistaxis were among the more frequently reported side-effects; however, no differences in haemoptysis event rates were reported between treatment and placebo groups [59, 60, 68, 69]. In the most recent interim analysis of the ongoing open-label SOTERIA study evaluating long-term safety, tolerability and efficacy of sotatercept in participants with PAH, five cases of haemoptysis were reported (1.2% of the 426 participants) [70].

In CHD-PAH, there is a high prevalence of pulmonary aneurysms and thrombogenic materials (*e.g.* vascular/valve prostheses), which might provoke *in situ* thrombosis and consecutive pulmonary embolism [18] and can increase the risk of haemoptysis. On the histological level, multiple angiomatoid-like lesions have been observed in CHD-PAH (figure 3), particularly in the case of large ventricular septal defects, representing a specific anatomical structure at risk for haemoptysis [65].

**FIGURE 3:**
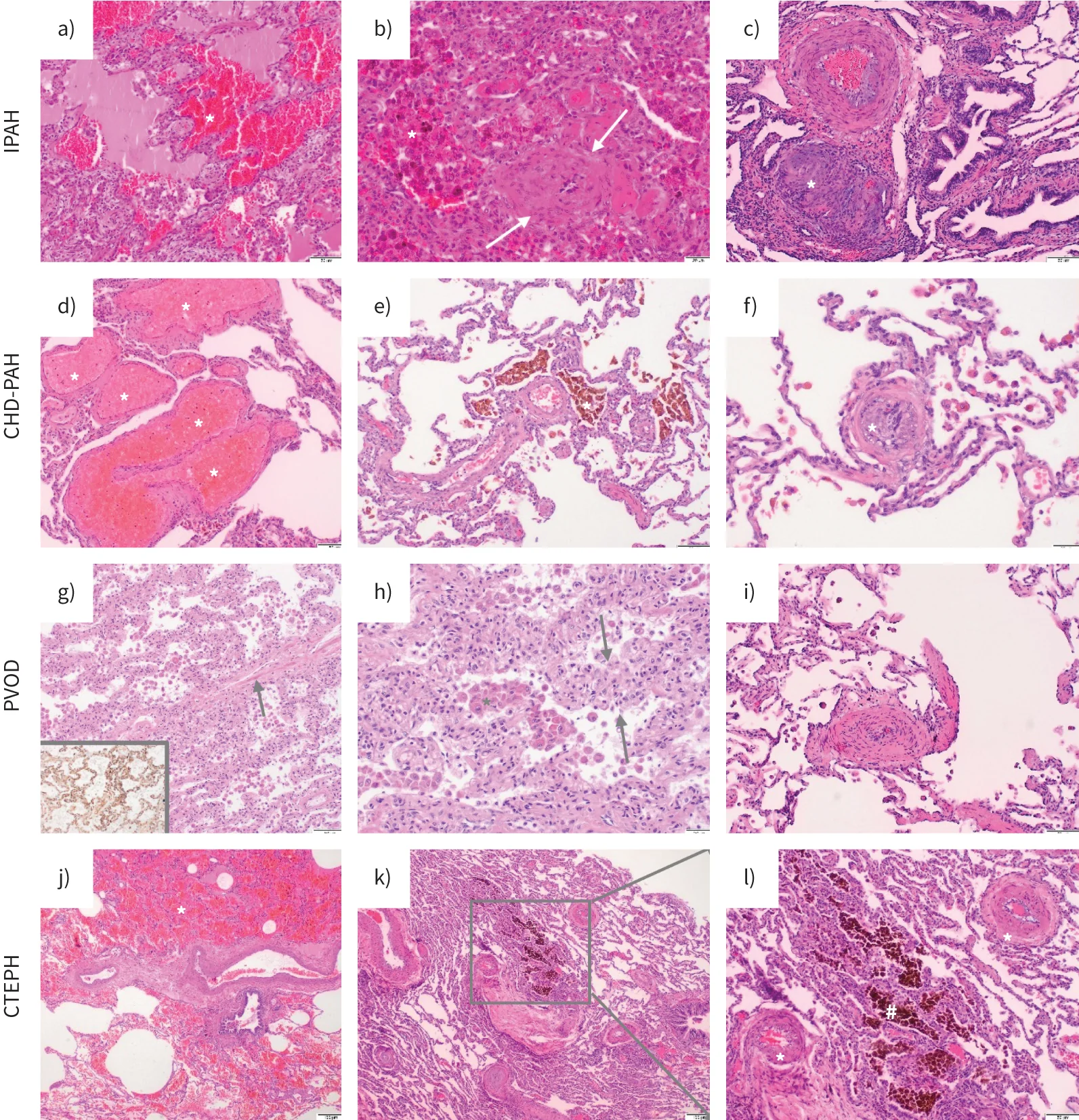
Histomorphological aspects. a–c) Idiopathic pulmonary arterial hypertension (IPAH) shows heterogenous coexistence of fresh (a) and old (b) haemorrhages as well as vascular remodelling with classic formation of plexiform lesions (c, indicated by asterisks). d–f) In vascular remodelling of congenital heart disease-associated pulmonary arterial hypertension (CHD-PAH), angiomatoid-like lesions (d, indicated by asterisks) and concentric pulmonary arterial remodelling (f, indicated by asterisk) have been observed, as well as intra-alveolar haemosiderin-laden macrophages as a sign that bleeding has occurred (e, indicated by asterisk). g–i) Pulmonary veno-occlusive disease (PVOD) is characterised by vascular remodelling predominantly affecting the postcapillary veins, with arterialisation of the venules (g, indicated by arrow) and pulmonary capillary haemangiomatosis with capillary proliferations in the area of the septa (g, inlay of anti-CD34 immunostaining; h, indicated by arrows) surrounded by haemosiderin-laden macrophages (h, indicated by asterisk). j–l) Heterogenous coexistence of fresh (j, indicated by asterisk) and old (k, l, indicated by #) haemorrhages in chronic thromboembolic pulmonary hypertension (CTEPH) with concentric vascular remodelling (l, indicated by asterisks). Scale bars: 100 µm (j, k), 50 µm (a, c–e, g, i, l) or 20 µm (b, f, h).

In PVOD, haemoptysis can occur *via* similar mechanisms as in PAH. One additional typical feature in these patients is an increased prevalence of haemosiderin-laden macrophages in bronchoalveolar lavage fluids caused by chronic occult alveolar haemorrhage due to postcapillary obstruction [26, 27].

Haemoptysis is a recognised complication in approximately 6% of patients with PAH. While the majority of haemoptysis episodes are mild and self-limiting, severe cases, although less frequent, can be life-threatening [57]. Even minor bleeding events should be considered warning signs in PAH, because small drops in oxygen saturation can trigger hypoxaemic vasoconstriction, escalating the risk of right heart strain and acute decompensation in patients with advanced PH.

Given that interventional treatment options are broadly available, surgical treatment options such as lobectomy or pneumonectomy are reserved for refractory or recurrent life-threatening pulmonary bleeding events owing to the associated high periprocedural risk and mortality [71].

There are few data on the incidence and presentation of pulmonary bleeding in PAH subgroups. For example, in an octennial observational study on 93 patients with CHD-PAH, hospitalisation due to haemoptysis was reported in about 5% of patients [39], with variable bleeding severity ranging from mild haemoptysis to massive haemorrhage. Accordingly, the clinical presentation depends on the severity of bleeding and can range from an innocuous taste of iron or bloody-tinged sputum to haemorrhagic shock and acute respiratory failure [72]. For patients with structural abnormalities predisposing to central pulmonary artery dissection or rupture, haemoptysis can occur along with cardiac tamponade [73].

CTA is the diagnostic method of choice in patients with PAH presenting with haemoptysis, often allowing for localisation of the bleeding source and assessment of bleeding extension and vascular morphology [21, 22]. However, even if vascular abnormalities are identified, these may not necessarily constitute the site of bleeding. In the absence of significant bronchial and non-bronchial systemic arterial supply on CTA and suspected pulmonary artery abnormality as the cause, computed tomography pulmonary angiography (CTPA) is the next step. In addition, CTPA can provide treatment guidance in case interventional therapies are required.

Although bronchoscopy may help identify the source of bleeding, its diagnostic use in PAH-associated haemoptysis is less common because CT imaging is usually sufficient [74].

In cases of moderate-to-severe haemoptysis, interventional BAE constitutes the first-line treatment, with an immediate clinical success rate of 70–99% [75]. Its efficacy in the treatment of PAH-associated haemoptysis, primarily related to CHD-PAH, has been reported in several case reports and small series [24, 25, 76, 77].

Although a number of studies have shown the feasibility of bronchoscopic techniques for managing haemoptysis events in patients with PAH, its use is less common owing to the convincing safety profile and increasing availability of targeted interventional angiography-guided therapies.

In patients with haemorrhagic diathesis, administration of clotting factors, antifibrinolytic agents like tranexamic acid [78, 79] and platelets might become necessary depending on coagulation status, fibrinolytic activity and blood count. In addition, existing anticoagulant or antiplatelet therapies should be individually evaluated for consideration of temporary or permanent discontinuation [80].

In patients with a history of pulmonary bleeding, prevention of recurrent bleeding events is critical. In cases of recurring moderate-to-severe haemoptysis or recurrent episodes of mild haemoptysis despite optimised medical treatment, embolisation of enlarged bronchial arteries is recommended by current European Respiratory Society (ERS)/European Society of Cardiology (ESC) guidelines and evaluation for lung transplantation should be considered [21, 22].

The literature describing mortality rates in patients with PAH who experience haemoptysis is scarce. Cantu *et al*. [81] reported an excessively high 28-day and 1-year mortality of 31% and 54%, respectively, whereas in general PAH patient cohorts the impact of haemoptysis events on mortality is perceived to be substantially lower. Long-term follow-up is essential due to the risk of recurrent bleeding, particularly in the setting of PAH progression.

Over the last few decades, advances in PAH treatment and management of bleeding complications have translated to significantly reduced morbidity and mortality in patients with Eisenmenger syndrome. While early studies ranked pulmonary haemorrhage as the leading cause of death [40, 82], Hjortshøj *et al*. [37] demonstrated that it had a substantially lower contribution to all-cause mortality in more recent cohorts. The risk of relapsing haemoptysis in patients with CHD-PAH is hard to estimate because the evidence is currently limited to patients with Eisenmenger syndrome, in whom recurrent haemoptysis has been observed in 17–20% of cases [42, 83, 84].

## Haemoptysis in other forms of pulmonary hypertension

### Chronic thromboembolic pulmonary hypertension

Chronic thromboembolic pulmonary hypertension (CTEPH) is characterised by chronic occlusion of pulmonary arteries, resulting in progressive increases in PAP and PVR, persistent ischaemia and development of bronchopulmonary collaterals. Owing to this aberrant vascularisation, haemoptysis is a relatively common complication in CTEPH, with reported incidence rates ranging from 4.7% to 28.3% [85–88]. The severity of haemoptysis appears to correlate with disease progression [89]. In addition, bronchial artery hypertrophy has been observed in many patients with CTEPH and is associated with an increased risk of pulmonary haemorrhage, although a recent study suggested that this adaptation to chronic hypoxia may improve gas exchange [90].

Imaging studies have revealed extensive bronchopulmonary collaterals as a result of, and an attempt to compensate for, impaired capillary blood flow, representing anatomical structures at increased risk for bleeding [90–92]. Collaterals between coronary and pulmonary arteries have been demonstrated in 10.8–22.9% of patients [93–95]. The propensity for extensive collateralisation and bleeding in patients with CTEPH has been further substantiated by a histopathological study [96].

Furthermore, CTEPH is associated with coagulopathies (antiphospholipid syndrome being the most common one) and other conditions such as myeloproliferative neoplasms, which can be associated with recurring thromboembolism, which in turn can provoke haemoptysis events [97–102].

In CTEPH, there is a correlation between the occurrence of pulmonary bleeding events and exposure to therapeutic anticoagulation. Lifelong anticoagulation represents the therapeutic backbone in these patients [21, 22]. The most commonly used anticoagulants for preventing recurrent thromboembolism in CTEPH are vitamin K antagonists and direct oral anticoagulants [103]. A large multicentric observational study showed a similar exposure-adjusted occurrence of haemoptysis events in patients with CTEPH receiving either vitamin K antagonists or direct oral anticoagulants, with incidence rates of 2.8% and 2.5%, respectively [104].

The therapeutic strategy for patients with CTEPH depends on the localisation of thromboembolic lesions. While pulmonary endarterectomy (PEA) is the treatment of choice for operable patients with central to segmental lesions, balloon pulmonary angioplasty (BPA), an interventional treatment modality that aims to revascularise occluded parts of the pulmonary vasculature, is indicated for inoperable patients and patients with recurrent or persistent PH following PEA [105]. The method aims to restore the pulmonary circulation by targeting predominantly segmental and subsegmental lesions. Haemoptysis is a common complication of BPA, occurring in 5.6–10% of procedures [106–110]. Indeed, procedural and post-interventional complications include vascular injury due to wire perforation, and lung injury with haemoptysis and/or hypoxia [21, 22]. Pulmonary haemorrhage is also a potential complication of PEA, usually occurring during reperfusion at the time of separation from the cardiopulmonary bypass [111].

PAH-approved therapies are available for patients with non-operable CTEPH or persistent/recurrent CTEPH following PEA. Riociguat, a soluble guanylate cyclase stimulator, is the most widely recommended pharmacological therapy approved in Europe for this indication. Overall, riociguat has demonstrated a favourable safety profile, as shown in large registry studies and meta-analyses [112, 113]. However, caution is required when prescribing riociguat to patients with a history of clinically significant haemoptysis according to the manufacturer's summary of product characteristics [114]. Similarly, a phase 2 study investigating the endothelin receptor antagonist macitentan in patients with inoperable CTEPH showed no evidence for the occurrence of haemoptysis events [115]. Finally, subcutaneous treprostinil has been investigated in patients with CTEPH in a randomised controlled phase 3 trial. Treprostinil is known to impair platelet aggregation through adenylate cyclase stimulation [116] and transformation of pulmonary arterial endoperoxides [117], although platelet counts usually remain unaffected. In this trial, serious adverse events leading to hospitalisation were reported in 18% of patients; however, the exact incidence of haemoptysis was not itemised [118]. In the original phase 3 trial investigating subcutaneous treprostinil in patients with PAH, three out of 233 patients who received treprostinil developed self-limiting gastrointestinal haemorrhage episodes, while pulmonary haemorrhage was not reported [119].

Because haemoptysis events can occasionally result from therapeutic interventions in CTEPH, they can often be anticipated and promptly treated. Although massive haemorrhage can occur, the vast majority of bleeding events in patients with CTEPH are mild and self-limiting [120].

CTPA is the gold standard for diagnosis in patients with CTEPH presenting with haemoptysis, allowing for comprehensive cardiopulmonary imaging, localisation of bleeding, visualisation of collateral circuits and planning of interventional therapies.

For patients with life-threatening haemoptysis, the application of emergency measures mirrors those described in patients with PAH. Once airway protection and cardiopulmonary stabilisation have been achieved, the patient should be emergently referred for interventional angiography to embolise culprit vessels and structures at risk of recurrent pulmonary bleeding, such as hypertrophied bronchial arteries and systemic-to-pulmonary collaterals [75, 120, 121]. In case of recurrent haemoptysis and failure of BAE, an emergency revascularisation strategy with PEA may be considered in select patients.

For the subgroup of patients with CTEPH who undergo BPA, different tools for the prediction of post-interventional haemoptysis have been suggested. Ikeda *et al*. [122] reported occlusive lesions assessed *via* post-interventional CT scans as independent predictors of procedure-related complications, including haemoptysis. Sugimoto *et al*. [123] demonstrated the applicability of left, right and main pulmonary artery indices for this purpose. These tools can be used for individual risk assessment. Based on the results of the RACE trial, which showed a higher frequency of lung injury after BPA in patients not receiving riociguat prior to the intervention, the ESC/ERS guidelines state that medical therapy should be considered prior to BPA in patients with CTEPH who are candidates for this intervention [21, 22, 124].

From a prognosis standpoint, there is no robust evidence to date demonstrating a clear impact of haemoptysis on mortality or long-term outcomes in patients with CTEPH.

### Takayasu arteritis

Takayasu arteritis is a rare large vessel vasculitis involving predominantly the aorta and large central arteries. Pulmonary artery involvement is less common, with the reported incidence ranging widely from 0% to 66% [125–127]. Haemoptysis events have been reported in 1.8–42.2% of patients with Takayasu arteritis [128–131] and occurs more frequently in patients with pulmonary artery involvement and associated PH [127, 129, 132]. PH prevalence in Takayasu arteritis ranges from 10% to 50% [127, 130]; in these patients, haemoptysis events can be massive and potentially life-threatening.

The pathophysiology of Takayasu arteritis is complex and driven by infiltrative vascular inflammation [133]. Affected arteries are exposed to a profound granulomatous inflammatory process that can lead to stenosis, occlusion and aneurysms. In addition, the disease is frequently accompanied by *in situ* thrombosis and PH, often classified as Group 4 PH owing to pulmonary artery obstructions [127, 134].

Haemoptysis events are common in patients with Takayasu arteritis, particularly in those with PH, where it occurs in over one third of patients [130]. These events can be massive and therefore perilous for affected patients [135–138]. In that setting, haemoptysis events seem to be a consequence of affected pulmonary arteries with chronically eroded walls and an increased intravascular pressure. Indeed, the majority of pulmonary bleeding events originate from pulmonary arteries, while bleeding from bronchial arteries is a rare event in Takayasu arteritis [135].

As in other forms of PH, CTPA is a valuable tool for assessing the source and degree of bleeding. However, because pulmonary bleeding events frequently present as massive haemorrhage stemming from large pulmonary artery segments affected by the disease, the origin of bleeding is typically localised to more proximal airways. Therefore, bronchoscopic interventions may be of greater relevance in Takayasu arteritis compared to other forms of PH. Given that endotracheal intubation is often required in massive pulmonary haemorrhage events, bronchoscopy can easily be applied and may offer significant diagnostic and therapeutic advantages in these situations [138].

The therapeutic strategy in patients with Takayasu arteritis and acute pulmonary haemoptysis events should be guided by the bleeding severity. In mild haemoptysis events, primary interventional transcatheter procedures can be applied to identify pulmonary or systemic culprit vessels with subsequent embolisation [135, 139]. In the event of massive haemorrhage, immediate emergency measures should be initiated as outlined in previous sections.

Given the complexity and high-risk nature of these patients, long-term follow-up is essential owing to the risk of disease progression and recurrent bleeding; however, robust data evaluating the impact of haemoptysis on survival and long-term outcomes remain limited.

## Haemoptysis in other chronic pulmonary vascular diseases

### Hereditary haemorrhagic telangiectasia

Hereditary haemorrhagic telangiectasia (HHT), also known as Osler­­–Weber–Rendu disease, is an autosomal-dominant genetic disorder that occurs in approximately one in 5000 individuals worldwide [140]. The disease is characterised by abnormal neoangiogenesis causing telangiectasias and AVMs, which can lead to various clinical manifestations, including epistaxis and gastrointestinal bleeding. Pulmonary bleeding has been reported to occur in 4–20% of patients with HHT [9, 141–143], although these numbers may be overestimated owing to selection and publication biases.

HHT is primarily caused by mutations in two genes: endoglin (*ENG*) and activin receptor-like kinase 1 (*ACVRL1*), also termed ALK1. Pulmonary arteriovenous malformations (PAVMs) affect 50–80% of patients with *ENG* mutations and 5–35% of patients with *ACVRL1* mutations [9–13]. PAVMs are abnormal connections between pulmonary arteries and veins, bypassing the capillary system. Besides contributing to hypoxaemia and paradoxical embolisation, PAVMs also increase the risk of haemoptysis. Tracheobronchial telangiectasias may also cause haemoptysis, albeit less commonly than PAVMs.

Pulmonary bleeding in HHT can range from mild haemoptysis to massive, life-threatening haemorrhage with significant blood loss, respiratory distress and hypoxaemia. Patients with known HHT who develop haemoptysis should be promptly evaluated for PAVMs, because these are the most likely source of bleeding. Haemoptysis in HHT can also rarely be seen alongside spontaneous haemothorax due to rupture of subpleural PAVMs, particularly in pregnant women [10, 141].

The diagnosis of pulmonary bleeding in HHT requires a high index of suspicion, particularly in patients with a known history of the disease or those presenting with characteristic symptoms. Imaging studies are crucial for diagnosis. Although PAVMs can frequently be identified on non-contrast CT scans [9], contrast-enhanced CT remains the gold standard for diagnosis, particularly in the setting of haemoptysis and/or prior to embolotherapy [144]. CT can provide detailed images of the vascular anatomy, allowing for the identification of AVMs and other vascular abnormalities (figure 4), while pulmonary angiography is restricted to therapeutic interventions. Bronchoscopy is usually not required but may be employed to localise the bleeding site, especially when the CT scan does not show obvious PAVMs and tracheobronchial telangiectasias are suspected.

**FIGURE 4:**
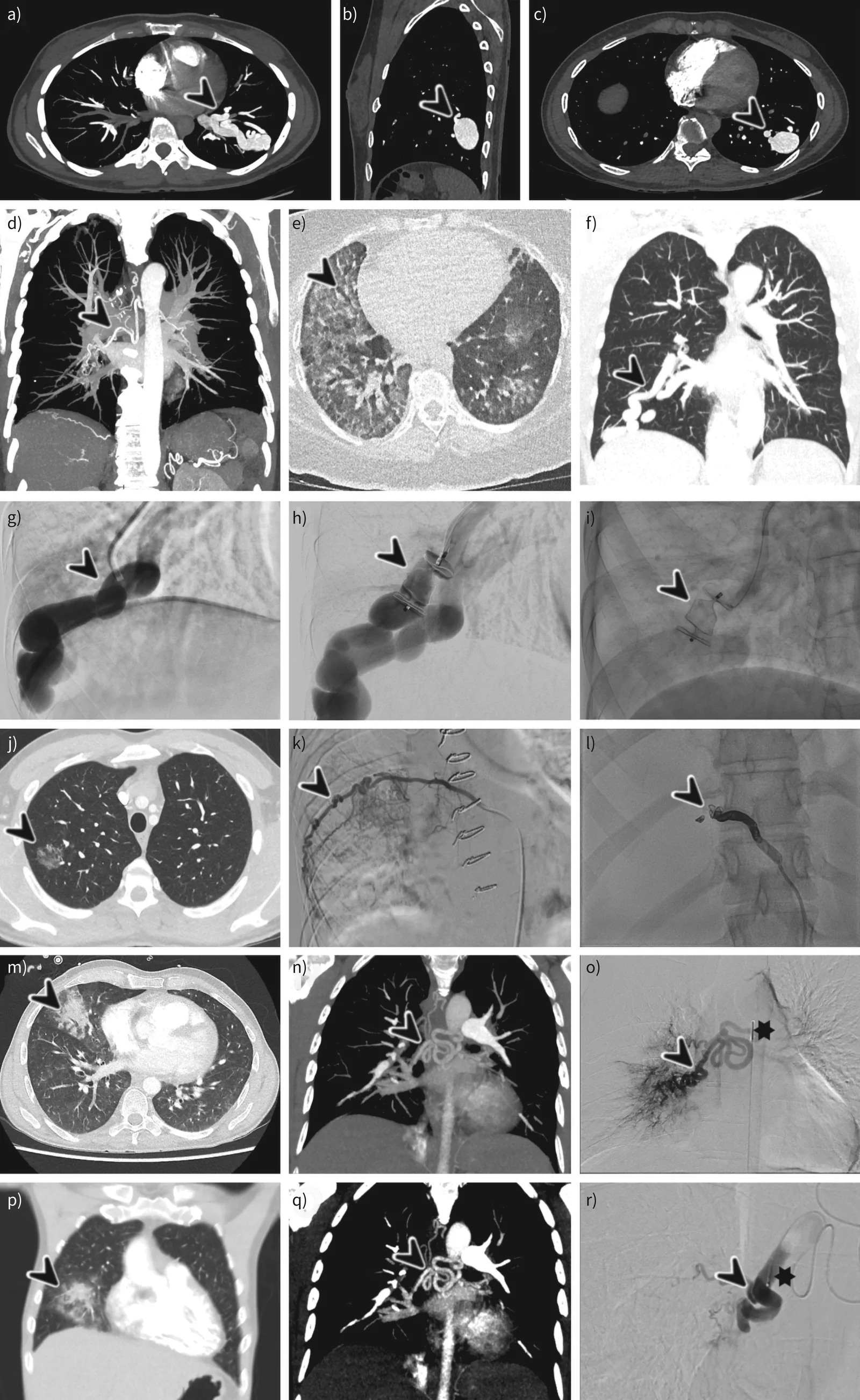
Imaging aspects. a–c) Computed tomography pulmonary angiography (CTPA) demonstrating a huge pulmonary arteriovenous malformation (AVM) (arrows) in a patient with hereditary haemorrhagic telangiectasia (HHT) in axial (a, c) and sagittal (b) planes. d) CTPA with arterial phase contrast showing emphasised bronchial artery hypertrophy in a patient with chronic thromboembolic pulmonary hypertension (CTEPH). The arrow indicates an enlarged bronchial artery. e) Axial CTPA plane showing pulmonary haemorrhage (arrow) in a patient with congenital heart disease-associated pulmonary arterial hypertension (CHD-PAH). f–i) Identification on CTPA (f) and angiography of an interventional occlusion by vascular plug insertion (g–i) of a large AVM in a patient with HHT. Arrows in f and g show the AVM, arrows in h and i point to the vascular plugging system. j–l) Identification on CTPA (j) and angiography of interventional coiling of a pulmonary haemorrhage from intercostal artery collaterals in a patient with CTEPH. Arrows indicate the bleeding site (j), the intercostal artery collaterals (k) and the inserted coils (l). m–r) Correlative CTPA images of a patient with CHD-PAH with pulmonary haemorrhage (arrows in m and p). n, q) CTPA images showing extensive bronchopulmonary collaterals as potential bleeding sources. o) Contrast medium injected *via* a diagnostic catheter (asterisk) shows bleeding from a hypertrophied bronchial artery (arrow). r) Contrast medium injected *via* a microcatheter (asterisk) showing successful embolisation of the target vessel.

Finally, transthoracic contrast echocardiography can both identify pulmonary shunting and be used for regular screening (usually every 2–3 years) to monitor for the development of PAVMs in patients with previously normal chest CT scans [145].

The role of medical interventions in the management of pulmonary bleeding in patients with HHT is limited, and evidence is largely lacking. Tranexamic acid, an antifibrinolytic agent, can be administered as adjunctive therapy, either orally or intravenously, but the evidence for its effectiveness comes mainly from epistaxis and gastrointestinal bleeding [146, 147]. The same is true for bevacizumab, a monoclonal antibody against VEGF [148, 149], and the calcineurin inhibitor tacrolimus [150]. None of these drugs are approved for control of bleeding in patients with HHT.

For definitive treatment, embolisation of PAVMs is the preferred approach [140, 143, 145, 151, 152]. Embolisation has been shown to be highly effective in controlling bleeding and preventing recurrence. Collateralisation or recanalisation of PAVMs following treatment may occur [153–155], necessitating long-term clinical and imaging follow-up. CT should be performed 6–12 months after embolisation and then approximately every 3–5 years. In rare cases in which embolisation is not possible or if there are multiple or inaccessible PAVMs, surgical resection may be considered.

For individuals with asymptomatic PAVMs, embolisation therapy can also be considered to prevent complications, with most centres embolising lesions with a feeding artery diameter of at least 2–3 mm [9].

*ACVRL1* and, more rarely, *ENG* mutations may lead to heritable PAH [21, 22]. In that setting, PAH and PAVMs may coexist, leading to difficult discussion of PAVM embolisation in patients with marked elevations in PVR. Indeed, severe PAH is generally considered a contraindication to performing PAVM embolisation because of the risk of worsening haemodynamic status. However, given the significant risk of rupture, paradoxical embolism and haemoptysis and the lack of data regarding the evolution of PAP after embolisation in PAH, PAVM embolisation should not be absolutely contraindicated and might be considered in patients with stable PAH [156]. Similarly, the use of bevacizumab has not been tested in patients with HHT with established PAH. There is no evidence of a causal association between bevacizumab and PAH in adults. However, bevacizumab may act as a secondary trigger in predisposed individuals, warranting continued pharmacovigilance, especially in patients carrying germline mutations increasing the risk of PAH [157].

With appropriate treatment, the prognosis for patients with HHT and pulmonary bleeding can be favourable. However, long-term follow-up is essential owing to the risk of recurrent bleeding and the development of new PAVMs. Regular monitoring with imaging studies and clinical evaluation is recommended because previously minor PAVMs may grow or embolised lesions may be reperfused [153]. Genetic counselling and testing may be advised for patients and their families to identify at-risk individuals and provide early intervention.

### Behcet's disease

Behcet's disease is a chronic, multisystem inflammatory disorder characterised by recurrent oral and genital ulcers, uveitis and skin lesions [158]. Pulmonary involvement, although less common, can lead to serious complications, including pulmonary bleeding. This condition often results from pulmonary vasculitis and the formation of pulmonary artery aneurysms (PAAs). Behcet's disease has been reported from almost all areas in the world but is most common in Japan, Iran and the Mediterranean countries [159]. Pulmonary vascular involvement is seen in less than 5% of patients, with PAAs and pulmonary vascular thrombosis being the most common pulmonary manifestations [137, 160–164].

The exact cause of Behcet's disease is unknown, but it is believed to involve an abnormal immune response, potentially triggered by environmental factors in genetically predisposed individuals. Vascular inflammation is a key feature, affecting arteries and veins of all sizes. In the lungs, this inflammation can lead to *in situ* thrombosis and the formation of PAAs, which are prone to rupture and may cause significant bleeding.

These patients will typically present with symptoms such as haemoptysis, which can range from mild to massive. Other symptoms may include cough, chest pain and dyspnoea. Diagnosis relies on clinical suspicion because there are no established biomarkers for the diagnosis of Behcet's disease. Imaging studies are crucial, with CTPA being the preferred modality to identify PAAs and assess the extent of vascular involvement. Bronchoscopy may also be used to localise the bleeding source.

Management of pulmonary bleeding in Behcet's disease focuses on controlling the acute haemorrhage and treating the underlying vasculitis. Endovascular techniques such as embolisation are often employed to control bleeding from PAAs. Immunosuppressive therapies, including corticosteroids and agents like cyclophosphamide or rituximab, are crucial to control the underlying inflammatory process and prevent recurrence [164].

### Hughes­–Stovin syndrome

HSS is a rare and potentially life-threatening disorder characterised by the presence of multiple PAAs and recurrent thrombophlebitis [19, 165]. It shares clinical features with Behcet's disease, and is sometimes considered a variant or subset of that condition [19, 20]. Pulmonary bleeding is one of the most severe complications associated with HSS, often resulting from the rupture of PAAs. In a review of 57 patients with HSS, haemoptysis was the initial disease presentation in 24 patients (42%), 53 patients (93%) developed haemoptysis during the course of the disease, and 12 patients (21%) had fatal pulmonary bleeding [166].

The exact aetiology of HSS is unknown, but it is thought to involve an abnormal immune response leading to inflammation and subsequent aneurysm formation in the pulmonary arteries. The inflammation causes the vessel walls to weaken and form aneurysms, which are at high risk for rupture and significant haemorrhage. Recurrent thrombophlebitis associated with HSS further complicates the vascular pathology, increasing the risk of thrombotic or embolic events [19, 20]. Pulmonary bleeding in HSS commonly presents with haemoptysis, which can vary from mild to severe and life-threatening. Other associated symptoms may include cough, chest pain, fever and dyspnoea.

As in Behcet's disease, there are no biomarkers specific for HSS. Hence, the diagnosis of HSS relies on imaging studies to identify PAAs and thrombotic lesions. As with other PVDs, CTPA is the diagnostic modality of choice for visualising aneurysms, assessing the extent of pulmonary involvement and identifying aneurysmal wall enhancement characteristic of HSS [20, 165, 167, 168]. Magnetic resonance imaging and conventional angiography can also be useful in certain cases. Laboratory tests to exclude other vasculitic and thrombotic conditions are also essential [169, 170].

Management of pulmonary bleeding in HSS is complex and requires a multidisciplinary approach. Acute treatment focuses on stabilising the patient and controlling haemorrhage, which may involve supportive measures such as oxygen, blood transfusions and mechanical ventilation if necessary. Endovascular procedures such as embolisation or stent-graft placement are often used to manage aneurysms and prevent further bleeding. Long-term management involves immunosuppressive therapy to control the underlying inflammatory process and prevent recurrence. Corticosteroids, along with other immunosuppressive agents like cyclophosphamide or azathioprine, are commonly employed [19, 20, 165].

**Points for clinical practice**

- Haemoptysis is commonly associated with chronic PVDs, contributing to significant morbidity and mortality in certain patient subsets.
- An individualised approach to diagnosis and management in this population is essential, guided by bleeding severity, anatomical localisation, vascular morphology and a nuanced understanding of the underlying condition.

**Questions for future research**

- Further research is needed to explore the complex pathophysiological mechanisms driving the development of haemoptysis in these patients, to improve diagnostic strategies, to optimise interventional treatment and to improve anticoagulation management.

## Supplementary material

**Please note:** supplementary material is not edited by the Editorial Office, and is uploaded as it has been supplied by the author. Supplementary material ERR-0055-2026.SUPPLEMENT

## References

[1] Yoon YC, Lee KS, Jeong YJ, et al. Hemoptysis: bronchial and nonbronchial systemic arteries at 16-detector row CT. Radiology 2005; 234: 292–298. doi:10.1148/radiol.2341032079 15550375

[2] Khalil A, Parrot A, Nedelcu C, et al. Severe hemoptysis of pulmonary arterial origin: signs and role of multidetector row CT angiography. Chest 2008; 133: 212–219. doi:10.1378/chest.07-1159 17989162

[3] Rabinovitch M. Pulmonary hypertension: pathophysiology as a basis for clinical decision making. J Heart Lung Transplant 1999; 18: 1041–1053. doi:10.1016/S1053-2498(99)00015-7 10598727

[4] Diller GP, Gatzoulis MA. Pulmonary vascular disease in adults with congenital heart disease. Circulation 2007; 115: 1039–1050. doi:10.1161/CIRCULATIONAHA.105.592386 17325254

[5] Savai R, Pullamsetti SS, Kolbe J, et al. Immune and inflammatory cell involvement in the pathology of idiopathic pulmonary arterial hypertension. Am J Respir Crit Care Med 2012; 186: 897–908. doi:10.1164/rccm.201202-0335OC 22955318

[6] Stacher E, Graham BB, Hunt JM, et al. Modern age pathology of pulmonary arterial hypertension. Am J Respir Crit Care Med 2012; 186: 261–272. doi:10.1164/rccm.201201-0164OC PMC3886716 22679007

[7] Ghigna MR, Guignabert C, Montani D, et al. *BMPR2* mutation status influences bronchial vascular changes in pulmonary arterial hypertension. Eur Respir J 2016; 48: 1668–1681. doi:10.1183/13993003.00464-2016 27811071

[8] Montani D, Lechartier B, Girerd B, et al. An emerging phenotype of pulmonary arterial hypertension patients carrying *SOX17* variants. Eur Respir J 2022; 60: 2200656. doi:10.1183/13993003.00656-2022 PMC10436756 35618278

[9] Circo S, Gossage JR. Pulmonary vascular complications of hereditary haemorrhagic telangiectasia. Curr Opin Pulm Med 2014; 20: 421–428. doi:10.1097/MCP.0000000000000076 25032812

[10] Cottin V, Dupuis-Girod S, Lesca G, et al. Pulmonary vascular manifestations of hereditary hemorrhagic telangiectasia (Rendu-Osler disease). Respiration 2007; 74: 361–378. doi:10.1159/000103205 17641482

[11] Bossler AD, Richards J, George C, et al. Novel mutations in *ENG* and *ACVRL1* identified in a series of 200 individuals undergoing clinical genetic testing for hereditary hemorrhagic telangiectasia (HHT): correlation of genotype with phenotype. Hum Mutat 2006; 27: 667–675. doi:10.1002/humu.20342 16752392

[12] Kjeldsen AD, Møller TR, Brusgaard K, et al. Clinical symptoms according to genotype amongst patients with hereditary haemorrhagic telangiectasia. J Intern Med 2005; 258: 349–355. doi:10.1111/j.1365-2796.2005.01555.x 16164574

[13] Lesca G, Olivieri C, Burnichon N, et al. Genotype–phenotype correlations in hereditary hemorrhagic telangiectasia: data from the French-Italian HHT network. Genet Med 2007; 9: 14–22. doi:10.1097/GIM.0b013e31802d8373 17224686

[14] Lill MC, Perloff JK, Child JS. Pathogenesis of thrombocytopenia in cyanotic congenital heart disease. Am J Cardiol 2006; 98: 254–258. doi:10.1016/j.amjcard.2006.01.083 16828603

[15] Henriksson P, Värendh G, Lundström NR. Haemostatic defects in cyanotic congenital heart disease. Br Heart J 1979; 41: 23–27. doi: 10.1136/hrt.41.1.23 PMC482008 426953

[16] Westbury SK, Lee K, Reilly-Stitt C, et al. High haematocrit in cyanotic congenital heart disease affects how fibrinogen activity is determined by rotational thromboelastometry. Thromb Res 2013; 132: e145–e151. doi:10.1016/j.thromres.2013.07.006 23906940

[17] Jensen AS, Johansson PI, Bochsen L, et al. Fibrinogen function is impaired in whole blood from patients with cyanotic congenital heart disease. Int J Cardiol 2013; 167: 2210–2214. doi:10.1016/j.ijcard.2012.06.019 22727966

[18] Broberg C, Ujita M, Babu-Narayan S, et al. Massive pulmonary artery thrombosis with haemoptysis in adults with Eisenmenger's syndrome: a clinical dilemma. Heart 2004; 90: e63. doi:10.1136/hrt.2004.039198 PMC1768531 15486107

[19] Khalid U, Saleem T. Hughes–Stovin syndrome. Orphanet J Rare Dis 2011; 6: 15. doi:10.1186/1750-1172-6-15 PMC3082226 21489283

[20] Ordu B, Aslan M, Ozguler Y, et al. Pulmonary artery involvement due to Behçet's syndrome and Hughes–Stovin syndrome: a comparative study. Clin Exp Rheumatol 2024; 42: 2021–2031. doi:10.55563/clinexprheumatol/t3i6xc 38197189

[21] ESC/ERS Scientific Document Group, Humbert M, Kovacs G, et al. 2022 ESC/ERS guidelines for the diagnosis and treatment of pulmonary hypertension. Eur Heart J 2022; 43: 3618–3731. doi:10.1093/eurheartj/ehac237 36017548

[22] ESC/ERS Scientific Document Group, Humbert M, Kovacs G, et al. 2022 ESC/ERS guidelines for the diagnosis and treatment of pulmonary hypertension. Eur Respir J 2023; 61: 2200879. doi:10.1183/13993003.00879-2022 36028254

[23] Baroutidou A, Arvanitaki A, Hatzidakis A, et al. Haemoptysis in pulmonary arterial hypertension associated with congenital heart disease: insights on pathophysiology, diagnosis and management. J Clin Med 2022; 11: 633. doi:10.3390/jcm11030633 PMC8836348 35160084

[24] Ulutaş Z, Kuloğlu HE, Kutlu R, et al. Successful bronchial artery embolization after stabilization with nitric oxide for the treatment of haemoptysis in a patient with Eisenmenger syndrome. Turk Kardiyol Dern Ars 2022; 50: 459–462. doi:10.5543/tkda.2022.21304 35983652

[25] Mamas MA, Clarke B, Mahadevan VS. Embolisation of systemic-to-pulmonary collaterals in patients with the Eisenmenger reaction presenting with haemoptysis. Cardiol Young 2008; 18: 528–531. doi:10.1017/S1047951108002680 18752715

[26] Rabiller A, Jaïs X, Hamid A, et al. Occult alveolar haemorrhage in pulmonary veno-occlusive disease. Eur Respir J 2006; 27: 108–113. doi:10.1183/09031936.06.00054105 16387942

[27] Lederer H, Muggli B, Speich R, et al. Haemosiderin-laden sputum macrophages for diagnosis in pulmonary veno-occlusive disease. PLoS One 2014; 9: e115219. doi:10.1371/journal.pone.0115219 PMC4264865 25501010

[28] Price LC, Dimopoulos K, Marino P, et al. The CRASH report: emergency management dilemmas facing acute physicians in patients with pulmonary arterial hypertension. Thorax 2017; 72: 1035–1045. doi:10.1136/thoraxjnl-2016-209725 28904006

[29] Ong TH, Eng P. Massive hemoptysis requiring intensive care. Intensive Care Med 2003; 29: 317–320. doi:10.1007/s00134-002-1553-6 12594593

[30] Gagnon S, Quigley N, Dutau H, et al. Approach to hemoptysis in the modern era. Can Respir J 2017; 2017: 1565030. doi:10.1155/2017/1565030 PMC5752991 29430203

[31] Sehgal IS, Dhooria S, Agarwal R, et al. Use of a flexible cryoprobe for removal of tracheobronchial blood clots. Respir Care 2015; 60: e128–e131. doi:10.4187/respcare.03861 25805837

[32] Valipour A, Kreuzer A, Koller H, et al. Bronchoscopy-guided topical hemostatic tamponade therapy for the management of life-threatening hemoptysis. Chest 2005; 127: 2113–2118. doi:10.1378/chest.127.6.2113 15947328

[33] Dweik RA, Stoller JK. Role of bronchoscopy in massive hemoptysis. Clin Chest Med 1999; 20: 89–105. doi:10.1016/S0272-5231(05)70129-5 10205720

[34] Davidson K, Shojaee S. Managing massive hemoptysis. Chest 2020; 157: 77–88. doi:10.1016/j.chest.2019.07.012 31374211

[35] Engelfriet PM, Duffels MG, Möller T, et al. Pulmonary arterial hypertension in adults born with a heart septal defect: the Euro Heart Survey on adult congenital heart disease. Heart 2007; 93: 682–687. doi:10.1136/hrt.2006.098848 PMC1955187 17164490

[36] Arvanitaki A, Giannakoulas G, Baumgartner H, et al. Eisenmenger syndrome: diagnosis, prognosis and clinical management. Heart 2020; 106: 1638–1645. doi:10.1136/heartjnl-2020-316665 32690623

[37] Hjortshøj CMS, Kempny A, Jensen AS, et al. Past and current cause-specific mortality in Eisenmenger syndrome. Eur Heart J 2017; 38: 2060–2067. doi:10.1093/eurheartj/ehx201 28430906

[38] CHALLENGE Investigators, Ntiloudi D, Apostolopoulou S, et al. Hospitalisations for heart failure predict mortality in pulmonary hypertension related to congenital heart disease. Heart 2019; 105: 465–469. doi:10.1136/heartjnl-2018-313613 30269081

[39] Schuuring MJ, van Riel AC, Vis JC, et al. New predictors of mortality in adults with congenital heart disease and pulmonary hypertension: midterm outcome of a prospective study. Int J Cardiol 2015; 181: 270–276. doi:10.1016/j.ijcard.2014.11.222 25535690

[40] Daliento L, Somerville J, Presbitero P, et al. Eisenmenger syndrome. Factors relating to deterioration and death. Eur Heart J 1998; 19: 1845–1855. doi:10.1053/euhj.1998.1046 9886728

[41] Broberg CS, Ujita M, Prasad S, et al. Pulmonary arterial thrombosis in Eisenmenger syndrome is associated with biventricular dysfunction and decreased pulmonary flow velocity. J Am Coll Cardiol 2007; 50: 634–642. doi:10.1016/j.jacc.2007.04.056 17692749

[42] Saha A, Balakrishnan KG, Jaiswal PK, et al. Prognosis for patients with Eisenmenger syndrome of various aetiology. Int J Cardiol 1994; 45: 199–207. doi:10.1016/0167-5273(94)90166-X 7960265

[43] Hascoet S, Fournier E, Jaïs X, et al. Outcome of adults with Eisenmenger syndrome treated with drugs specific to pulmonary arterial hypertension: a French multicentre study. Arch Cardiovasc Dis 2017; 110: 303–316. doi:10.1016/j.acvd.2017.01.006 28286190

[44] Cantor WJ, Harrison DA, Moussadji JS, et al. Determinants of survival and length of survival in adults with Eisenmenger syndrome. Am J Cardiol 1999; 84: 677–681. doi:10.1016/S0002-9149(99)00415-4 10498138

[45] Montani D, Price LC, Dorfmuller P, et al. Pulmonary veno-occlusive disease. Eur Respir J 2009; 33: 189–200. doi:10.1183/09031936.00090608 19118230

[46] Neubert L, Borchert P, Stark H, et al. Molecular profiling of vascular remodeling in chronic pulmonary disease. Am J Pathol 2020; 190: 1382–1396. doi:10.1016/j.ajpath.2020.03.008 32275906

[47] Montani D, O'Callaghan DS, Savale L, et al. Pulmonary veno-occlusive disease: recent progress and current challenges. Respir Med 2010; 104: Suppl. 1, S23–S32. doi:10.1016/j.rmed.2010.03.014 20456932

[48] Liang L, Ma G, Chen K, et al. *EIF2AK4* mutation in pulmonary veno-occlusive disease: a case report and review of the literature. Medicine (Baltimore) 2016; 95: e5030. doi:10.1097/MD.0000000000005030 PMC5265969 27684876

[49] Agrawal V, Costopoulos KA, Chowdhary M, et al. Extracorporeal membrane oxygenation as a treatment for branch pulmonary artery rupture following right heart catheterisation. Card Fail Rev 2022; 8: e03. doi:10.15420/cfr.2021.25 PMC8845036 35223086

[50] Villaverde RV, Vanhaebost J, Grabherr S, et al. Pulmonary artery rupture during Swan-Ganz catheterisation: a case report. Leg Med (Tokyo) 2014; 16: 76–80. doi:10.1016/j.legalmed.2013.11.003 24332120

[51] Atreya AR, Arora S, Valania G. Pulmonary artery rupture with pseudoaneurysm formation secondary to Swan-Ganz catheter balloon inflation. Acute Card Care 2015; 17: 77–79. doi:10.3109/17482941.2016.1174270 27283269

[52] Kearney TJ, Shabot MM. Pulmonary artery rupture associated with the Swan-Ganz catheter. Chest 1995; 108: 1349–1352. doi:10.1378/chest.108.5.1349 7587440

[53] Hiraki T, Kanazawa S, Mimura H, et al. Transcatheter embolization of pulmonary artery false aneurysm associated with primary pulmonary hypertension. Cardiovasc Intervent Radiol 2004; 27: 186–189. doi:10.1007/s00270-003-2722-x 15259821

[54] Khalil A, Parrot A, Fartoukh M, et al. Pulmonary artery occlusion with ethylene vinyl alcohol copolymer in patients with hemoptysis: initial experience in 12 cases. AJR Am J Roentgenol 2012; 198: 207–212. doi:10.2214/AJR.10.5370 22194499

[55] Grosse C, Grosse A. CT findings in diseases associated with pulmonary hypertension: a current review. Radiographics 2010; 30: 1753–1777. doi:10.1148/rg.307105710 21057119

[56] Grubstein A, Benjaminov O, Dayan DB, et al. Computed tomography angiography in pulmonary hypertension. Isr Med Assoc J 2008; 10: 117–120. 18432023

[57] Tio D, Leter E, Boerrigter B, et al. Risk factors for hemoptysis in idiopathic and hereditary pulmonary arterial hypertension. PLoS One 2013; 8: e78132. doi:10.1371/journal.pone.0078132 PMC3806771 24194909

[58] Grynblat J, Bogaard HJ, Eyries M, et al. Pulmonary vascular phenotype identified in patients with *GDF2* (*BMP9*) or *BMP10* variants: an international multicentre study. Eur Respir J 2024; 63: 2301634. doi:10.1183/13993003.01634-2023 38514094

[59] Humbert M, McLaughlin V, Gibbs JSR, et al. Sotatercept for the treatment of pulmonary arterial hypertension: PULSAR open-label extension. Eur Respir J 2023; 61: 2201347. doi:10.1183/13993003.01347-2022 PMC9816418 36041750

[60] Hoeper MM, Badesch DB, Ghofrani HA, et al. Phase 3 trial of sotatercept for treatment of pulmonary arterial hypertension. N Engl J Med 2023; 388: 1478–1490. doi:10.1056/NEJMoa2213558 36877098

[61] Savale L, Tu L, Normand C, et al. Effect of sotatercept on circulating proteomics in pulmonary arterial hypertension. Eur Respir J 2024; 64: 2401483. doi:10.1183/13993003.01483-2024 PMC11525346 39227073

[62] McDonald DM. Angiogenesis and remodeling of airway vasculature in chronic inflammation. Am J Respir Crit Care Med 2001; 164: S39–S45. doi:10.1164/ajrccm.164.supplement_2.2106065 11734465

[63] Chun JY, Morgan R, Belli AM. Radiological management of hemoptysis: a comprehensive review of diagnostic imaging and bronchial arterial embolization. Cardiovasc Intervent Radiol 2010; 33: 240–250. doi:10.1007/s00270-009-9788-z 20058006

[64] Marshall TJ, Jackson JE. Vascular intervention in the thorax: bronchial artery embolization for haemoptysis. Eur Radiol 1997; 7: 1221–1227. doi:10.1007/s003300050279 9377505

[65] Haroutunian LM, Neill CA. Pulmonary complications of congenital heart disease: hemoptysis. Am Heart J 1972; 84: 540–559. doi:10.1016/0002-8703(72)90479-6 4672656

[66] Rubin LJ, Galiè N, Grimminger F, et al. Riociguat for the treatment of pulmonary arterial hypertension: a long-term extension study (PATENT-2). Eur Respir J 2015; 45: 1303–1313. doi:10.1183/09031936.00090614 25614164

[67] McLaughlin V V, Benza RL, Rubin LJ, et al. Addition of inhaled treprostinil to oral therapy for pulmonary arterial hypertension: a randomized controlled clinical trial. J Am Coll Cardiol 2010; 55: 1915–1922. doi:10.1016/j.jacc.2010.01.027 20430262

[68] Humbert M, McLaughlin VV, Badesch DB, et al. Sotatercept in patients with pulmonary arterial hypertension at high risk for death. N Engl J Med 2025; 392: 1987–2000. doi:10.1056/NEJMoa2415160 40167274

[69] McLaughlin VV, Hoeper MM, Badesch DB, et al. Sotatercept for pulmonary arterial hypertension within the first year after diagnosis. N Engl J Med 2025; 393: 1599–1611. doi:10.1056/NEJMoa2508170 41025556

[70] Preston IR, Badesch D, Ghofrani HA, et al. A long-term follow-up study of sotatercept for treatment of pulmonary arterial hypertension: interim results of SOTERIA. Eur Respir J 2025; 66: 2401435. doi:10.1183/13993003.01435-2024 PMC12287605 39978862

[71] Ittrich H, Bockhorn M, Klose H, et al. The diagnosis and treatment of hemoptysis. Dtsch Arztebl Int 2017; 114: 371–381. doi:10.3238/arztebl.2017.0371 PMC5478790 28625277

[72] Roofthooft MT, Douwes JM, Vrijlandt EJ, et al. Frequency and prognostic significance of hemoptysis in pediatric pulmonary arterial hypertension. Am J Cardiol 2013; 112: 1505–1509. doi:10.1016/j.amjcard.2013.06.034 23972345

[73] Chulamanee P, Siriwattanaskul P, Vachirawongsakorn V, et al. Ruptured pulmonary artery aneurysm with prior dissection and persistent ductus arteriosus: autopsy case report. Cardiovasc Pathol 2024; 73: 107684. doi:10.1016/j.carpath.2024.107684 39134126

[74] Lundgren R, Häggmark S, Reiz S. Hemodynamic effects of flexible fiberoptic bronchoscopy performed under topical anesthesia. Chest 1982; 82: 295–299. doi:10.1378/chest.82.3.295 7105856

[75] Panda A, Bhalla AS, Goyal A. Bronchial artery embolization in hemoptysis: a systematic review. Diagn Interv Radiol 2017; 23: 307–317. doi:10.5152/dir.2017.16454 PMC5508955 28703105

[76] Baroutidou A, Arvanitaki A, Mouratoglou SA, et al. Successful bronchial artery embolization in a patient with Eisenmenger syndrome and recurrent haemoptysis. Int J Cardiol Congenit Heart Dis 2022; 8: 100386. doi:10.1016/j.ijcchd.2022.100386 PMC11657923 39712043

[77] Rasciti E, Sverzellati N, Silva M, et al. Bronchial artery embolization for the treatment of haemoptysis in pulmonary hypertension. Radiol Med 2017; 122: 257–264. doi:10.1007/s11547-016-0714-6 28025781

[78] Tsai YS, Hsu LW, Wu MS, et al. Effects of tranexamic acid on hemoptysis: a systematic review and meta-analysis of randomized controlled trials. Clin Drug Investig 2020; 40: 789–797. doi:10.1007/s40261-020-00946-y 32661913

[79] Lee HN, Park HS, Hyun D, et al. Combined therapy with bronchial artery embolization and tranexamic acid for hemoptysis. Acta Radiol 2021; 62: 610–618. doi:10.1177/0284185120933984 32551802

[80] Baumgartner H, Bonhoeffer P, De Groot NM, et al. ESC Guidelines for the management of grown-up congenital heart disease (new version 2010). Eur Heart J 2010; 31: 2915–2957. doi:10.1093/eurheartj/ehq249 20801927

[81] Cantu J, Wang D, Safdar Z. Clinical implications of haemoptysis in patients with pulmonary arterial hypertension. Int J Clin Pract Suppl 2012; 177: 5–12. doi:10.1111/ijcp.12004 PMC4001782 22943513

[82] Wood P. The Eisenmenger syndrome or pulmonary hypertension with reversed central shunt. I. Br Med J 1958; 2: 701–709. doi:10.1136/bmj.2.5098.701 PMC2026671 13572874

[83] Jensen AS, Iversen K, Vejlstrup NG, et al. Images in cardiovascular medicine. Pulmonary artery thrombosis and hemoptysis in Eisenmenger syndrome. Circulation 2007; 115: e632–e634. doi:10.1161/CIRCULATIONAHA.107.695312 17576876

[84] Montani D, Achouh L, Dorfmüller P, et al. Pulmonary veno-occlusive disease: clinical, functional, radiologic, and hemodynamic characteristics and outcome of 24 cases confirmed by histology. Medicine (Baltimore) 2008; 87: 220–233. doi:10.1097/MD.0b013e31818193bb 18626305

[85] Simonneau G, Torbicki A, Dorfmüller P, et al. The pathophysiology of chronic thromboembolic pulmonary hypertension. Eur Respir Rev 2017; 26: 160112. doi:10.1183/16000617.0112-2016 PMC9488693 28356405

[86] Yang S, Wang J, Kuang T, et al. Efficacy and safety of bronchial artery embolization on hemoptysis in chronic thromboembolic pulmonary hypertension: a pilot prospective cohort study. Crit Care Med 2019; 47: e182–e189. doi:10.1097/CCM.0000000000003578 PMC6407824 30531186

[87] Xie WM, Wang J, Zhang S, et al. [Clinical characteristics of patients with chronic thromboembolic pulmonary hypertension]. Zhonghua Yi Xue Za Zhi 2019; 99: 3461–3465. 10.3760/cma.j.issn.0376-2491.2019.44.003 31826562

[88] Garcia-Alonso C, Gerardin B, Moisson L, et al. Traitement du cœur pulmonaire post-embolique chez le sujet âgé [Treatment of chronic thromboembolic pulmonary hypertension in elderly patients]. Ann Cardiol Angeiol (Paris) 2018; 67: 482–488. doi:10.1016/j.ancard.2018.09.014 30463686

[89] Li Y, Liang L, Guo D, et al. Right ventricular function predicts adverse clinical outcomes in patients with chronic thromboembolic pulmonary hypertension: a three-dimensional echocardiographic study. Front Med (Lausanne) 2021; 8: 697396. doi:10.3389/fmed.2021.697396 PMC8419302 34497813

[90] Loureiro CMC, Ferreira EVM, Oliveira RKF, et al. Gas exchange dynamics and responses to exercise in chronic thromboembolic pulmonary hypertension. Respir Physiol Neurobiol 2026; 339: 104490. doi:10.1016/j.resp.2025.104490 40975194

[91] Kanchi M, Nair HC, Natarajan P, et al. Management of intrapulmonary hemorrhage in patients undergoing pulmonary thrombo-endarterectomy. Ann Card Anaesth 2021; 24: 384–388. doi:10.4103/aca.ACA_191_20 PMC8404590 34269276

[92] Sun W, Ota H, Sato H, et al. Systemic-pulmonary collateral supply associated with clinical severity of chronic thromboembolic pulmonary hypertension: a study using intra-aortic computed tomography angiography. Eur Radiol 2022; 32: 7668–7679. doi:10.1007/s00330-022-08768-6 PMC9668953 35420297

[93] Goyal A, Avery R, Cuttica MJ, et al. Case report: multimodality imaging of unusual coronary to pulmonary collaterals in chronic thromboembolic pulmonary hypertension. Front Cardiovasc Med 2023; 10: 1204736. doi:10.3389/fcvm.2023.1204736 PMC10285476 37363090

[94] Kepez A, Mutlu B, Paudel A, et al. Prevalence of coronary artery to pulmonary artery collaterals in patients with chronic thromboembolic pulmonary hypertension: retrospective analysis from a single center. Thorac Cardiovasc Surg 2018; 66: 180–186. doi:10.1055/s-0036-1584572 27326914

[95] Lee NS, Blanchard DG, Knowlton KU, et al. Prevalence of coronary artery-pulmonary artery collaterals in patients with chronic thromboembolic pulmonary hypertension. Pulm Circ 2015; 5: 313–321. doi:10.1086/681225 PMC4449242 26064456

[96] Yamaki S, Ando M, Fukumoto Y, et al. Histopathological examination by lung biopsy for the evaluation of operability and postoperative prognosis in patients with chronic thromboembolic pulmonary hypertension. Circ J 2014; 78: 476–482. doi:10.1253/circj.CJ-13-1016 24284920

[97] Marsh JJ, Chiles PG, Liang NC, et al. Chronic thromboembolic pulmonary hypertension-associated dysfibrinogenemias exhibit disorganized fibrin structure. Thromb Res 2013; 132: 729–734. doi:10.1016/j.thromres.2013.09.024 24182551

[98] Morris TA, Marsh JJ, Chiles PG, et al. High prevalence of dysfibrinogenemia among patients with chronic thromboembolic pulmonary hypertension. Blood 2009; 114: 1929–1936. doi:10.1182/blood-2009-03-208264 PMC2738577 19420351

[99] Colorio CC, Martinuzzo ME, Forastiero RR, et al. Thrombophilic factors in chronic thromboembolic pulmonary hypertension. Blood Coagul Fibrinolysis 2001; 12: 427–432. doi:10.1097/00001721-200109000-00002 11555695

[100] Montani D, Thoré P, Mignard X, et al. Clinical phenotype and outcomes of pulmonary hypertension associated with myeloproliferative neoplasms: a population-based study. Am J Respir Crit Care Med 2023; 208: 600–612. doi:10.1164/rccm.202210-1941OC 37311222

[101] Guilpain P, Montani D, Damaj G, et al. Pulmonary hypertension associated with myeloproliferative disorders: a retrospective study of ten cases. Respiration 2008; 76: 295–302. doi:10.1159/000112822 18160817

[102] Momoi M, Katsumata Y, Kunimoto H, et al. Clonal hematopoiesis in chronic thromboembolic pulmonary hypertension. J Am Heart Assoc 2024; 13: e035498. 10.1161/JAHA.124.035498 PMC11681603 39604025

[103] Escal J, Poenou G, Delavenne X, et al. Tailoring oral anticoagulant treatment in the era of multi-drug therapies for PAH and CTEPH. Blood Rev 2024; 68: 101240. doi:10.1016/j.blre.2024.101240 39245607

[104] Humbert M, Simonneau G, Pittrow D, et al. Oral anticoagulants (NOAC and VKA) in chronic thromboembolic pulmonary hypertension. J Heart Lung Transplant 2022; 41: 716–721. doi:10.1016/j.healun.2022.02.002 35305871

[105] Hoeper MM, Madani MM, Nakanishi N, et al. Chronic thromboembolic pulmonary hypertension. Lancet Respir Med 2014; 2: 573–582. doi:10.1016/S2213-2600(14)70089-X 24898750

[106] Li X, Zhang Y, Jin Q, et al. Development and validation of a nomogram for balloon pulmonary angioplasty-related complications in patients with chronic thromboembolic pulmonary hypertension. Rev Cardiovasc Med 2023; 24: 72. doi:10.31083/j.rcm2403072 PMC11263984 39077502

[107] Kennedy MK, Kennedy SA, Tan KT, et al. Balloon pulmonary angioplasty for chronic thromboembolic pulmonary hypertension: a systematic review and meta-analysis. Cardiovasc Intervent Radiol 2023; 46: 5–18. doi:10.1007/s00270-022-03323-8 36474104

[108] Kawakami T, Matsubara H, Shinke T, et al. Balloon pulmonary angioplasty versus riociguat in inoperable chronic thromboembolic pulmonary hypertension (MR BPA): an open-label, randomised controlled trial. Lancet Respir Med 2022; 10: 949–960. doi:10.1016/S2213-2600(22)00171-0 35926544

[109] Karyofyllis P, Demerouti E, Giannakoulas G, et al. Balloon pulmonary angioplasty in patients with chronic thromboembolic pulmonary hypertension in Greece: data from the Hellenic Pulmonary Hypertension Registry. J Clin Med 2022; 11: 2211. doi:10.3390/jcm11082211 PMC9028480 35456303

[110] Olsson KM, Wiedenroth CB, Kamp JC, et al. Balloon pulmonary angioplasty for inoperable patients with chronic thromboembolic pulmonary hypertension: the initial German experience. Eur Respir J 2017; 49: 1602409. doi:10.1183/13993003.02409-2016 28596435

[111] Jenkins DP, Martinez G, Salaunkey K, et al. Perioperative management in pulmonary endarterectomy. Semin Respir Crit Care Med 2023; 44: 851–865. doi:10.1055/s-0043-1770123 37487525

[112] Ying M, Song J, Gu S, et al. Efficacy and safety of riociguat in the treatment of chronic thromboembolic pulmonary arterial hypertension: a meta-analysis. Medicine (Baltimore) 2021; 100: e26211. doi:10.1097/MD.0000000000026211 PMC8183702 34087896

[113] Ghofrani HA, Gomez Sanchez MA, Humbert M, et al. Riociguat treatment in patients with chronic thromboembolic pulmonary hypertension: final safety data from the EXPERT registry. Respir Med 2021; 178: 106220. doi:10.1016/j.rmed.2020.106220 33540340

[114] European Medicines Agency. Adempas: Summary of Product Characteristics. Available from: https://ec.europa.eu/health/documents/community-register/2018/20180307140104/anx_140104_en.pdf 2019.

[115] Ghofrani HA, Simonneau G, D'Armini AM, et al. Macitentan for the treatment of inoperable chronic thromboembolic pulmonary hypertension (MERIT-1): results from the multicentre, phase 2, randomised, double-blind, placebo-controlled study. Lancet Respir Med 2017; 5: 785–794. doi:10.1016/S2213-2600(17)30305-3 28919201

[116] Gryglewski RJ. Prostaglandins, platelets, and atherosclerosis. CRC Crit Rev Biochem 1980; 7: 291–338. doi:10.3109/10409238009105464 6771102

[117] Moncada S, Gryglewski R, Bunting S, et al. An enzyme isolated from arteries transforms prostaglandin endoperoxides to an unstable substance that inhibits platelet aggregation. Nature 1976; 263: 663–665. doi:10.1038/263663a0 802670

[118] Sadushi-Kolici R, Jansa P, Kopec G, et al. Subcutaneous treprostinil for the treatment of severe non-operable chronic thromboembolic pulmonary hypertension (CTREPH): a double-blind, phase 3, randomised controlled trial. Lancet Respir Med 2019; 7: 239–248. doi:10.1016/S2213-2600(18)30367-9 30477763

[119] Simonneau G, Barst RJ, Galie N, et al. Continuous subcutaneous infusion of treprostinil, a prostacyclin analogue, in patients with pulmonary arterial hypertension: a double-blind, randomized, placebo-controlled trial. Am J Respir Crit Care Med 2002; 165: 800–804. doi:10.1164/ajrccm.165.6.2106079 11897647

[120] Clements W, Venn G, McGiffin D, et al. chronic thromboembolic pulmonary hypertension (CTEPH) and massive hemoptysis: the rationale for bronchial artery embolization. Respir Med 2022; 195: 106784. doi:10.1016/j.rmed.2022.106784 35232634

[121] Reesink HJ, van Delden OM, Kloek JJ, et al. Embolization for hemoptysis in chronic thromboembolic pulmonary hypertension: report of two cases and a review of the literature. Cardiovasc Intervent Radiol 2007; 30: 136–139. doi:10.1007/s00270-005-0382-8 17086459

[122] Ikeda N, Kubota S, Okazaki T, et al. The predictors of complications in balloon pulmonary angioplasty for chronic thromboembolic pulmonary hypertension. Catheter Cardiovasc Interv 2019; 93: E349–E356. doi:10.1002/ccd.28133 30773792

[123] Sugimoto K, Nakazato K, Sakamoto N, et al. Pulmonary artery diameter predicts lung injury after balloon pulmonary angioplasty in patients with chronic thromboembolic pulmonary hypertension. Int Heart J 2017; 58: 584–588. doi:10.1536/ihj.16-365 28701673

[124] Jaïs X, Brenot P, Bouvaist H, et al. Balloon pulmonary angioplasty *versus* riociguat for the treatment of inoperable chronic thromboembolic pulmonary hypertension (RACE): a multicentre, phase 3, open-label, randomised controlled trial and ancillary follow-up study. Lancet Respir Med 2022; 10: 961–971. doi:10.1016/S2213-2600(22)00214-4 35926542

[125] Hotchi M. Pathological studies on Takayasu arteritis. Heart Vessels Suppl 1992; 7: 11–17. doi:10.1007/BF01744538 1360954

[126] Sharma BK, Jain S, Radotra BD. An autopsy study of Takayasu arteritis in India. Int J Cardiol 1998; 66: Suppl. 1, S85–S90. doi:10.1016/S0167-5273(98)00155-7 9951807

[127] He Y, Lv N, Dang A, et al. Pulmonary artery involvement in patients with Takayasu arteritis. J Rheumatol 2020; 47: 264–272. doi:10.3899/jrheum.190045 31092716

[128] Makhzoum JP, Grayson PC, Ponte C, et al. Pulmonary involvement in primary systemic vasculitides. Rheumatology (Oxford) 2021; 61: 319–330. doi:10.1093/rheumatology/keab325 PMC8857773 33788906

[129] Xi X, Du J, Liu J, et al. Pulmonary artery involvement in Takayasu arteritis: a retrospective study in Chinese population. Clin Rheumatol 2021; 40: 635–644. doi:10.1007/s10067-020-05271-5 32643055

[130] Wang J, Lei K, Li J, et al. Analysis of clinical features and prognostic factors in Takayasu arteritis involving pulmonary hypertension: a retrospective study. Medicine (Baltimore) 2024; 103: e37992. doi:10.1097/MD.0000000000037992 PMC11062657 38701260

[131] Toledano K, Guralnik L, Lorber A, et al. Pulmonary arteries involvement in Takayasu's arteritis: two cases and literature review. Semin Arthritis Rheum 2011; 41: 461–470. doi:10.1016/j.semarthrit.2011.06.001 21803399

[132] Liao H, Zhang N, Pan L, et al. Predictors for pulmonary artery involvement in Takayasu arteritis and its cluster analysis. Arthritis Res Ther 2023; 25: 9. doi:10.1186/s13075-022-02987-4 PMC9840297 36639641

[133] Pugh D, Karabayas M, Basu N, et al. Large-vessel vasculitis. Nat Rev Dis Primers 2022; 7: 93. doi:10.1038/s41572-021-00327-5 PMC9115766 34992251

[134] Jiang X, Zhu YJ, Zhou YP, et al. Clinical features and survival in Takayasu's arteritis-associated pulmonary hypertension: a nationwide study. Eur Heart J 2021; 42: 4298–4305. doi:10.1093/eurheartj/ehab599 34506618

[135] Imamura R, Hayashi K, Sada KE, et al. Hemoptysis originating from the bronchial artery in Takayasu arteritis with ulcerative colitis. Intern Med 2019; 58: 293–295. doi:10.2169/internalmedicine.1463-18 PMC6378154 30146600

[136] Rocha MP, Guntupalli KK, Moise KJ Jr, et al. Massive hemoptysis in Takayasu's arteritis during pregnancy. Chest 1994; 106: 1619–1622. doi:10.1378/chest.106.5.1619 7956437

[137] Nasser M, Cottin V. The respiratory system in autoimmune vascular diseases. Respiration 2018; 96: 12–28. doi:10.1159/000486899 29975964

[138] Yang X, Ma W, Shi X, et al. Novel approach of rigid bronchoscopy concurrent with cesarean section and bronchial arterial embolism for patients with massive hemoptysis during pregnancy: case reports and literature review. Ann Transl Med 2021; 9: 582. doi:10.21037/atm-20-2502 PMC8105789 33987280

[139] Lopez AJ, Brady AJ, Jackson JE. Case report: therapeutic bronchial artery embolization in a case of Takayasu's arteritis. Clin Radiol 1992; 45: 415–417. doi:10.1016/S0009-9260(05)81005-0 1351435

[140] Al-Samkari H. Hereditary hemorrhagic telangiectasia: systemic therapies, guidelines, and an evolving standard of care. Blood 2021; 137: 888–895. doi:10.1182/blood.2020008739 33171488

[141] Ference BA, Shannon TM, White RI, et al. Life-threatening pulmonary hemorrhage with pulmonary arteriovenous malformations and hereditary hemorrhagic telangiectasia. Chest 1994; 106: 1387–1390. doi: 10.1378/chest.106.5.1387 7956388

[142] Faughnan ME, Granton JT, Young LH. The pulmonary vascular complications of hereditary haemorrhagic telangiectasia. Eur Respir J 2009; 33: 1186–1194. doi:10.1183/09031936.00061308 19407052

[143] Cottin V, Chinet T, Lavolé A, et al. Pulmonary arteriovenous malformations in hereditary hemorrhagic telangiectasia: a series of 126 patients. Medicine (Baltimore) 2007; 86: 1–17. doi:10.1097/MD.0b013e31802f8da1 17220751

[144] Saboo SS, Chamarthy M, Bhalla S, et al. Pulmonary arteriovenous malformations: diagnosis. Cardiovasc Diagn Ther 2018; 8: 325–337. doi:10.21037/cdt.2018.06.01 PMC6039795 30057879

[145] Faughnan ME, Mager JJ, Hetts SW, et al. Second international guidelines for the diagnosis and management of hereditary hemorrhagic telangiectasia. Ann Intern Med 2020; 173: 989–1001. doi:10.7326/M20-1443 32894695

[146] Gaillard S, Dupuis-Girod S, Boutitie F, et al. Tranexamic acid for epistaxis in hereditary hemorrhagic telangiectasia patients: a European cross-over controlled trial in a rare disease. J Thromb Haemost 2014; 12: 1494–1502. doi:10.1111/jth.12654 25040799

[147] Geisthoff UW, Seyfert UT, Kübler M, et al. Treatment of epistaxis in hereditary hemorrhagic telangiectasia with tranexamic acid – a double-blind placebo-controlled cross-over phase IIIB study. Thromb Res 2014; 134: 565–571. doi:10.1016/j.thromres.2014.06.012 25005464

[148] Al-Samkari H, Kasthuri RS, Parambil JG, et al. An international, multicenter study of intravenous bevacizumab for bleeding in hereditary hemorrhagic telangiectasia: the InHIBIT-Bleed study. Haematologica 2021; 106: 2161–2169. doi:10.3324/haematol.2020.261859 PMC8327711 32675221

[149] Iyer VN, Apala DR, Pannu BS, et al. Intravenous bevacizumab for refractory hereditary hemorrhagic telangiectasia-related epistaxis and gastrointestinal bleeding. Mayo Clin Proc 2018; 93: 155–166. doi:10.1016/j.mayocp.2017.11.013 29395350

[150] Hessels J, Kroon S, Boerman S, et al. Efficacy and safety of tacrolimus as treatment for bleeding caused by hereditary hemorrhagic telangiectasia: an open-label, pilot study. J Clin Med 2022; 11: 5280. doi:10.3390/jcm11185280 PMC9503120 36142926

[151] Mager JJ, Overtoom TT, Blauw H, et al. Embolotherapy of pulmonary arteriovenous malformations: long-term results in 112 patients. J Vasc Interv Radiol 2004; 15: 451–456. doi:10.1097/01.RVI.0000126811.05229.B6 15126654

[152] Faughnan ME, Palda VA, Garcia-Tsao G, et al. International guidelines for the diagnosis and management of hereditary haemorrhagic telangiectasia. J Med Genet 2011; 48: 73–87. doi:10.1136/jmg.2009.069013 19553198

[153] Milic A, Chan RP, Cohen JH, et al. Reperfusion of pulmonary arteriovenous malformations after embolotherapy. J Vasc Interv Radiol 2005; 16: 1675–1683. doi:10.1097/01.RVI.0000182163.25493.BB 16371535

[154] Shimohira M, Kawai T, Hashizume T, et al. Reperfusion rates of pulmonary arteriovenous malformations after coil embolization: evaluation with time-resolved MR angiography or pulmonary angiography. J Vasc Interv Radiol 2015; 26: 856–864.e1. doi:10.1016/j.jvir.2015.02.016 25851199

[155] Expert Panel on Vascular Imaging, Pillai AK, Steigner ML, Aghayev A, et al. ACR appropriateness criteria pulmonary arteriovenous malformation (PAVM): 2023 update. J Am Coll Radiol 2024; 21: S268–S285. doi:10.1016/j.jacr.2024.02.028 38823949

[156] Montani D, Price LC, Girerd B, et al. Fatal rupture of pulmonary arteriovenous malformation in hereditary haemorrhagic telangiectasis and severe PAH. Eur Respir Rev 2009; 18: 42–46. doi:10.1183/09059180.00011113 20956122

[157] Hlavaty A, Roustit M, Montani D, et al. Identifying new drugs associated with pulmonary arterial hypertension: a WHO pharmacovigilance database disproportionality analysis. Br J Clin Pharmacol 2022; 88: 5227–5237. doi:10.1111/bcp.15436 PMC9795981 35679331

[158] Fazaa A, Makhlouf Y, Ben Massoud F, et al. Behçet disease: epidemiology, classification criteria and treatment modalities. Expert Rev Clin Immunol 2024; 20: 1437–1448. doi:10.1080/1744666X.2024.2388693 39101633

[159] Celik S, Yazici Y, Sut N, et al. Pulmonary artery aneurysms in Behçet's syndrome: a review of the literature with emphasis on geographical differences. Clin Exp Rheumatol 2015; 33: 6 Suppl. 94, S54–S59. 26211653

[160] Hamuryudan V, Yurdakul S, Moral F, et al. Pulmonary arterial aneurysms in Behçet's syndrome: a report of 24 cases. Br J Rheumatol 1994; 33: 48–51. doi:10.1093/rheumatology/33.1.48 8162457

[161] Hamuryudan V, Er T, Seyahi E, et al. Pulmonary artery aneurysms in Behçet syndrome. Am J Med 2004; 117: 867–870. doi:10.1016/j.amjmed.2004.05.027 15589493

[162] Uzun O, Erkan L, Akpolat I, et al. Pulmonary involvement in Behçet's disease. Respiration 2008; 75: 310–321. doi:10.1159/000101954 17446699

[163] Uzun O, Akpolat T, Erkan L. Pulmonary vasculitis in Behcet disease: a cumulative analysis. Chest 2005; 127: 2243–2253. doi:10.1378/chest.127.6.2243 15947344

[164] Seyahi E, Melikoglu M, Akman C, et al. Pulmonary artery involvement and associated lung disease in Behçet disease: a series of 47 patients. Medicine (Baltimore) 2012; 91: 35–48. doi:10.1097/MD.0b013e318242ff37 22210555

[165] Sanduleanu S, Jansen T. Hughes–Stovin syndrome (HSS): current status and future perspectives. Clin Rheumatol 2021; 40: 4787–4789. doi:10.1007/s10067-021-05958-3 34655003

[166] Emad Y, Ragab Y, Kechida M, et al. A critical analysis of 57 cases of Hughes–Stovin syndrome (HSS). A report by the HSS International Study Group (HSSISG). Int J Cardiol 2021; 331: 221–229. doi:10.1016/j.ijcard.2021.01.056 33529654

[167] Ketchum ES, Zamanian RT, Fleischmann D. CT angiography of pulmonary artery aneurysms in Hughes–Stovin syndrome. AJR Am J Roentgenol 2005; 185: 330–332. doi:10.2214/ajr.185.2.01850330 16037501

[168] Emad Y, Ragab Y, Farber HW, et al. Pulmonary embolism *versus* pulmonary vasculitis in Hughes–Stovin syndrome: characteristic computed tomography pulmonary angiographic findings and diagnostic and therapeutic implications. HSS International Study Group. Thromb Res 2024; 239: 109040. doi:10.1016/j.thromres.2024.109040 38795561

[169] Cho SB, Yun M, Lee JH, et al. Detection of cardiovascular system involvement in Behçet's disease using fluorodeoxyglucose positron emission tomography. Semin Arthritis Rheum 2011; 40: 461–466. doi:10.1016/j.semarthrit.2010.05.006 20822800

[170] Denecke T, Staeck O, Amthauer H, et al. PET/CT visualises inflammatory activity of pulmonary artery aneurysms in Behçet disease. Eur J Nucl Med Mol Imaging 2007; 34: 970. doi:10.1007/s00259-007-0429-y 17447060

