# Supplementary material for: Haemoptysis in chronic pulmonary vascular disease: a narrative review
Source: Eur Respir Rev. 2026 Oct 21;35(182):260055. doi: 10.1183/16000617.0055-2026 (PMC13684417; doi:10.1183/16000617.0055-2026)
Supplement: Supplementary file 1 [file ERR-0055-2026.SUPPLEMENT.pdf]

## Supplementary table 1 – Haemoptysis events in major clinical studies of pulmonary arterial hypertension drugs

| Therapy | Source | Study design | Patients | Study duration | Target dose(s) of intervention Drug | Comparator | Baseline therapy (not including CCB) | Haemoptysis in study group | Haemoptysis in comparator |
|---|---|---|---|---|---|---|---|---|---|
| **Phosphodiesterase type 5 inhibitors** | | | | | | | | | |
| Sildenafil | SUPER-1 (2005)(1) | Randomized, double-blind, placebo-controlled trial | 278 | 12 weeks | 80mg PO TID (n=71)<br>40mg PO TID (n=67)<br>20mg PO TID (n=69) | Placebo (n=70) | None | 0/71 (80mg TID)<br>0/67 (40mg TID)<br>0/69 (20mg TID) | 0/70 |
| | SERAPH (2005)(2) | Randomized, double-blind, placebo-controlled trial | 26 | 16 weeks | 50mg PO TID (n=14) | Bosentan 125mg PO BID (n=12) | None | 0/14 | 1/12 |
| | Singh et al (2006)(3) | Randomized, double-blind, placebo-controlled crossover trial | 20 | 6 weeks (12 including crossover) | 100mg PO TID (n=10) | Placebo (n=10) | None | 0/10 | 0/10 |
| | PACES (2008)(4) | Randomized, double-blind, placebo-controlled trial | 267 | 16 weeks | 80mg PO TID (n=134) | Placebo (n=131) | Epoprostenol (267) | 0/134 | 1/131 |
| | Vizza et al (2017)(5) | Randomized, double-blind trial with open-label extension | 129 | 12 weeks (24 weeks include OLE) | 5mg PO TID (n=43)<br>1mg PO TID (n=41) | Sildenafil 20mg PO TID (n=45) | None | 0/43 (5mg PO TID)<br>0/41 (1mg PO TID) | 0/45 (20mg PO TID) |
| | AFFILIATE (2024)(6) | Randomized, double-blind trial | 385 | 137 weeks (mean) | 80mg PO TID (n=128) | Sildenafil 20mg PO TID (n=128)<br>Sildenafil 5 mg PO TID (n=129) | Ambrisentan (11)<br>Beraprost (4)<br>Bosentan (1)<br>Epoprostenol (7)<br>Iloprost (6)<br>Macitentan (26) | 0/128 (80mg PO TID) | 0/128 (20mg PO TID)<br>0/129 (5mg PO TID) |
| Tadalafil | PHIRST (2009)(7) | Randomized, double-blind, placebo-controlled trial | 405 | 16 weeks | 40mg PO daily (n=79)<br>20mg PO daily (n=82)<br>10mg PO daily (n=80)<br>2.5mg PO daily (n=82) | Placebo (n=82) | None (189)<br>Bosentan (216) | 0/323 (all doses of 2.5, 10, 20, 40) | 0/82 |
| | Zhuang et al (2014)(8) | Randomized, double-blind, placebo-controlled trial | 124 | 16 weeks | 40mg PO daily (n=60) | Placebo (n=64) | Ambrisentan (124) | 0/60 | 0/64 |
| **Soluble guanylate cyclase stimulators** | | | | | | | | | |
| Riociguat | PATENT-1 (2013)(9) | Randomized, double-blind, placebo-controlled trial | 443 | 12 weeks | 2.5mg PO TID (n=254)<br>1.5mg PO TID (n=63) | Placebo | None (221)<br>ERA (194)<br>PCA (28) | 2/254 (2.5mg PO TID)<br>0/63 (1.5mg PO TID) | 0/126 |
| | PATENT-2 (2015)(10) long-term extension study | Open-label extension of PATENT-1 | 396 | 95 weeks (mean) | 2.5mg PO TID (n=396) | None | Not reported | 16/231 (former 2.5mg PO TID)<br>2/56 (former 1.5mg PO TID)<br>7/109 (former placebo) | N/A |
| | PATENT PLUS (2015)(11) | Randomized, double-blind, placebo-controlled trial | 18 | 12 weeks | 2.5mg PO TID (n=12) | Placebo (n=6) | Sildenafil (18) | 0/12 | 0/6 |
| | REPLACE (2021)(12) | Open-label, randomized controlled trial | 225 | 24 weeks | 2.5mg PO TID (n=111) | Continue PDE5i (n=114) | PDE5i monotherapy (64) | 0/111 | 0/114 |

| | | | | | | | | | |
|---|---|---|---|---|---|---|---|---|---|
| | | | | | | | PDE5i + ERA (160) | | |
| MK-5475 | INSIGNIA-PAH (2024)(13) | Randomized, double-blind, placebo-controlled trial | 168 | 12 weeks | 32µg PO daily (n=61)<br>100µg PO daily (n=62)<br>380µg PO daily (n=62) | Placebo (n=41) | | 0/42 (32µg)<br>1/44 (100µg)<br>0/41 (380µg | 0/41 |
| **Endothelin Receptor Antagonists** | | | | | | | | | |
| Ambrisentan | ARIES 1/2 (2008)(14) | Randomized, double-blind, placebo-controlled trial | 393 | 12 weeks | 10mg PO daily (n=67)<br>5mg PO daily (n=130)<br>2.5mg PO daily (n=64) | Placebo (n=132) | None | 0/67 (10mg PO daily)<br>0/130 (5mg PO daily)<br>0/64 (2.5mg PO daily) | 0/132 |
| Bosentan | Bosentan (2001)(15) | Randomized, double-blind, placebo-controlled trial | 32 | 12 weeks | 125mg PO BID (n=21) | Placebo (n=11) | None | 0/21 | 0/11 |
| | BREATHE-1 (2002)(16) | Randomized, double-blind, placebo-controlled trial | 213 | 16 weeks in phase 1<br>28 weeks including phase 1 and 2 | 250mg PO BID (n=70)<br>125mg PO BID (n=74) | Placebo (n=69) | None | 0/70 (250mg PO BID) Note: 1 died of pulmonary haemorrhage in OLE study)<br>0/74 (125mg PO BID) | 0/69 |
| | EARLY (2008)(17) | Randomized, double-blind, placebo-controlled trial | 185 | 26 weeks | 125mg PO BID (n=93) | Placebo (n=92) | None (156) Sildenafil (29) | 0/93 | 0/92 |
| | COMPASS-2 (2015)(18) | Randomized, double-blind, placebo-controlled trial | 334 | 220 weeks (mean) | 125mg PO BID (n=159) | Placebo (n=175) | Sildenafil (334) | 0/159 | 0/175 |
| Macitentan | SERAPHIN (2013)(19) | Randomized, double-blind, placebo-controlled trial | 742 | 115 weeks (mean) | 10mg PO daily (n=242)<br>3mg PO daily (n=250) | Placebo (n=250) | None (268)<br>PDE5i (454)<br>Oral/inhaled PCA (40) | 0/242 (10mg PO daily)<br>0/250 (3mg PO daily) | 0/250 |
| Sitaxsentan | STRIDE-1 (2004)(20) | Randomized, double-blind, placebo-controlled trial | 178 | 12 weeks | 300mg PO daily (n=63)<br>100mg PO daily (n=55) | Placebo (n=60) | None | 0/63 (300mg PO daily)<br>0/56 (100mg PO daily) | 0/59 |
| | STRIDE-2 (2006)(21) | Randomized, double-blind, placebo-controlled trial | 247 | 18 weeks | 100mg PO daily (n=61)<br>50mg PO daily (n=62) | Open-label bosentan 125mg PO BID (n=60)<br>Placebo (n=62) | None | 0/61 (100mg PO daily)<br>0/62 (50mg PO daily) | 0/60 (bosentan 125mg PO BID)<br>0/62 (placebo) |
| **Prostacyclin Receptor Agonist** | | | | | | | | | |
| Selexipag | Simonneau et al (2012)(22) | Randomized, double-blind, placebo-controlled trial | 43 | 17 weeks | 800mcg PO BID (n=33) | Placebo (n=10) | ERA (16)<br>Sildenafil (12)<br>ERA + sildenafil (15) | 0/33 | 0/10 |
| | GRIPHON (2015)(23) | Randomized, double-blind, placebo-controlled trial | 1156 | 98.1 weeks (median) | 1600mcg PO BID (n=574) | Placebo (n=582) | None (236)<br>ERA (170)<br>PDE5i (374)<br>ERA + PDE5i (376) | 0/574 | 0/582 |
| | TRACE (2023)(24) | Randomized, double-blind, placebo-controlled trial | 108 | 24 weeks | 1600mcg PO BID (n=53) | Placebo (n=55) | ERA (2)<br>ERA + PDE5i or ERA + sGCs (106) | 0/53 | 0/55 |
| Ralinepag | Torres et al (2019)(25,26) | Randomized, double-blind, placebo-controlled trial | 61 | 22 weeks | 300mcg PO BID (n=40) | Placebo (n=21) | ERA (6)<br>PDE5i (19)<br>ERA + PDE5i (56)<br>ERA + sGCs (2) | 0/40<br>0/30 in OLE (median 153.4 weeks) | 0/21<br>0/15 in OLE (median 153.4 weeks) |

| | | | | | | | | | |
|---|---|---|---|---|---|---|---|---|---|
| **Prostacyclin analogues** | | | | | | | | | |
| Epoprostenol (IV) | Barst et al (1996) – (NEJM)(27) | Randomized, controlled, open-label study | 81 | 12 weeks | Mean maximal tolerated dose 9.2ng/kg/min (n=41) | Conventional therapy (n=40) | None | 0/41 | 0/40 |
| Treprostinil (oral) | FREEDOM-C1 (2012)(28) | Randomized, double-blind, placebo-controlled trial | 350 | 16 weeks | Mean maximal tolerated dose 3.0mg PO BID (n=174) | Placebo (n=176) | ERA (106)<br>PDE5i (88)<br>ERA + PDE5i (156) | 0/174 | 0/176 |
| | FREEDOM-C2 (2013)(29) | Randomized, double-blind, placebo-controlled trial | 310 | 16 weeks | Mean maximal tolerated dose 3.1mg PO BID (n=157) | Placebo (n=153) | ERA (53)<br>PDE5i (132)<br>ERA + PDE5i (125) | 0/157 | 0/153 |
| | FREEDOM-M (2013)(30) | Randomized, double-blind, placebo-controlled trial | 349 | 12 weeks | Mean maximal tolerated dose 3.6mg PO BID (n=233) | Placebo (n=116) | None | 0/233 | 0/116 |
| | FREEDOM-EV (2020)(31) | Randomized, double-blind, placebo-controlled trial | 690 | 58 weeks (median) | Median maximal tolerated dose 3.56mg PO TID (n=346) | Placebo (n=344) | PDE5i or sGC alone (494)<br>ERA alone (196) | 0/346 | 0/344 |
| Treprostinil (SC) | Simonneau et al (2002)(32) | Randomized, double-blind, placebo-controlled trial | 470 | 12 weeks | Mean maximal tolerated dose 9.3 ng/kg/min (n=233) | Placebo (n=236) | None | 0/233 | 0/236 |
| Treprostinil (inhaled) | TRIUMPH (2010)(33) | Randomized, double-blind, placebo-controlled trial | 235 | 12 weeks | Mean maximal tolerated daily dose 50mcg (n=115) | Placebo (n=120) | Bosentan (165)<br>Sildenafil (70) | 0/115<br>NB in FDA label* | 0/120 |
| Iloprost (inhaled) | AIR (2002)(34) | Randomized, double-blind, placebo-controlled trial | 203 | 12 weeks | Median maximal tolerated daily dose 30mcg (n=101) | Placebo (n=102) | None | Not reported in study; FDA label = 5 | Not reported in study; FDA label = 2 |
| | STEP (2006)(35) | Randomized, double-blind, placebo-controlled trial | 67 | 12 weeks | Mean maximal tolerated daily dose 26.8mcg (n=34) | Placebo (n=33) | Bosentan (67) | 0/34 | 0/33 |
| Beraprost | ALPHABET (2002)(36) | Randomized, double-blind, placebo-controlled trial | 130 | 12 weeks | Median dose 80mcg PO QID (n=65) | Placebo (n=65) | None | 0/65 | 0/65 |
| | Barst et al (2003)(37) | Randomized, double-blind, placebo-controlled trial | 116 | 12 months | Median dose 120mcg PO QID (n=60) | Placebo (n=56) | None | 0/60 | 0/56 |
| **Activin signalling inhibitors** | | | | | | | | | |
| Sotatercept | PULSAR (2021)(38,39) | Randomized, double-blind, placebo-controlled trial | 106 | 24 weeks with 18 month open-label extension | 0.7mg/kg SC q3 weeks (n=42)<br>0.3mg/kg SC q3 weeks (n=32) | Placebo (n=32) | PCA infusion (39)<br>Monotherapy (10)<br>Double therapy (25)<br>Triple therapy (59) | 0/42 at 0.7mg/kg; 0/36 during OLE<br>0/32 at 0.3mg/kg; 0/31 during OLE | 0/32; 0/32 during OLE |
| | STELLAR (2023)(40) | Randomized, double-blind, placebo-controlled trial | 323 | 24 weeks | 0.7mg/kg SC q3 weeks (n=163) | Placebo (n=160) | PCA infusion (129)<br>Monotherapy (13)<br>Double therapy (112)<br>Triple therapy (198) | 3/163 | 2/160 |
| | ZENITH (2025)(41) | Randomized, double-blind, placebo-controlled trial | 172 | 8.9 months (median) | 0.7mg/kg SC q3 weeks (n=86) | Placebo (n=86) | PCA infusion (102)<br>Double therapy (48)<br>Triple therapy (124) | 0/86 | 2/86 |
| | HYPERION (2025)(42) | Randomized, double-blind, placebo-controlled trial | 320 | 13.2 months (median) | 0.7mg/kg SC q3 weeks (n=160) | Placebo (n=160) | PCA infusion (53)<br>Double therapy (231)<br>Triple therapy (89) | 0/160 | 1/160 |
| **Tyrosine kinase inhibitors** | | | | | | | | | |
| Imatinib | Ghofrani et al (2010)(43) | Randomized, double-blind, placebo-controlled trial | 59 | 6 months | 400mg PO daily (n=28) | Placebo (n=31) | ERA (6)<br>Sildenafil (2)<br>PCA (3) | 1/28 | 0/31 |

| | | | | | | | | | |
|---|---|---|---|---|---|---|---|---|---|
| | | | | | | | ERA + PCA (4)<br>ERA + sildenafil (21)<br>Sildenafil + PCA (8)<br>ERA + sildenafil + PCA (14) | | |
| | IMPRES (2013)(44) | Randomized, double-blind, placebo-controlled trial | 202 | 24 weeks | 400mg PO daily (n=103) | Placebo (n=98) | ERA + PDE5 (59)<br>ERA + PCA (25)<br>PCA + PDE5i (34)<br>ERA + PCA + PDE5i (83) | 0/103 | 2/98 |
| Imatinib (inhaled) | IMPAHCT (NCT05036135); IMPAHCT-FUL (NCT05557942) (45) | Randomized, double-blind, placebo-controlled trial with open-label extension | 202 (186 in OLE) | 24 weeks | 70mg BID (n=51)<br>35mg BID (n=49)<br>10mg BID (n=50) | Placebo (n=52) | PCA infusion (74)<br>Triple therapy (117)<br>Double therapy (85) | 0/150;<br>3/186 during OLE | |
| Seralutinib (inhaled) | TORREY (2024)(46) | Randomized, double-blind, placebo-controlled trial | 86 | 24 weeks | 90mg BID (n=42) | Placebo (n=42) | Triple therapy (49)<br>Double therapy (34)<br>Monotherapy (3) | 1/44 | 1/42 |
| **Upfront combination therapy** | | | | | | | | | |
| Epoprostenol + bosentan | BREATHE-2 (2004)(47) | Randomized, double-blind, placebo-controlled trial | 33 | 16 weeks | Bosentan 125mg PO BID + epoprostenol 12-16ng/kg/min (n=22) | Epoprostenol alone (n=11) | None | 0/22 | 0/11 |
| Iloprost + bosentan | COMBI (2006)(48) | Randomized, controlled, open-label study | 40 | 12 weeks | Iloprost total daily dose 30mcg + bosentan 125mg PO BID (n=19) | Bosentan alone (n=21) | None | 1/19 | 0/21 |
| Ambrisentan + tadalafil | AMBITION (2015)(49) | Randomized, double-blind, placebo-controlled trial | 500 | 87 weeks (mean) | Ambrisentan 10mg PO daily + tadalafil 40mg PO daily (n=253) | Ambrisentan + placebo (n=126)<br>Tadalafil + placebo (n=121) | None | 0/253 | 0/247 |
| Macitentan + tadalafil + selexipag | TRITON (2021)(50) | Randomized, double-blind, placebo-controlled trial | 247 | 26 weeks | Macitentan 10mg PO daily + tadalafil 40mg PO daily + selexipag 1600mcg PO daily (n=123) | Macitentan + tadalafil (124) | None | 0/123 | 0/124 |
| Macitentan/ Tadalafil Single-Tablet Combination | A DUE (2024)(51) | Randomized, double-blind, placebo-controlled trial | 187 | 16 weeks | Macitenan 10mg/tadalafil 40mg single-tablet combination PO daily (n=108) | Macitentan (35)<br>Tadalafil (44) | ERA (32)<br>PDE5i (56) | 0/107 | 0/35 (macitentan)<br>0/44 (tadalafil) |

PDE5i = Phosphodiesterase type 5 inhibitor; sGC = soluble guanylyl cyclase stimulator; ERA = endothelin receptor antagonist; PCA = prostacyclin analogue, CCB = calcium channel blocker; OLE = open label extension

*FDA label for inhaled treprostinil: In a prospective, observational study comparing patients taking Tyvaso (958 patient-years of exposure) and a control group (treatment with other approved therapies for PAH; 1094 patient-years), Tyvaso was associated with a higher rate of cough (16.2 vs.

10.9 per 100 patient-years), throat irritation (4.5 vs. 1.2 per 100 pt-years), nasal discomfort (2.6 vs. 1.3 per 100 pt-years), and hemoptysis (2.5 vs. 1.3 per 100 pt-years) compared to the control group.

**Supplementary table 2 – Haemoptysis events in major clinical studies of drugs for specific pulmonary arterial hypertension aetiologies**

| Therapy | Source | Study design | Patients | Study duration | Target dose(s) of intervention drug | Comparator | Baseline therapy (not including CCB) | Haemoptysis in study group | Haemoptysis in comparator |
|---|---|---|---|---|---|---|---|---|---|
| **Systemic sclerosis** | | | | | | | | | |
| Epoprostenol (IV) | Epoprostenol SSc – Badesch et al (2000)(52) | Randomized, controlled, open-label study | 111 | 12 weeks | Mean or median maximal tolerated dose unspecified (n=56) | Conventional therapy (n=55) | None | 0/56 (2 had "haemorrhage" related to delivery system; not specified pulmonary) | 0/55 |
| **Eisenmenger syndrome** | | | | | | | | | |
| Bosentan | BREATHE-5 (2006)(53) | Randomized, double-blind, placebo-controlled trial | 54 | 16 weeks | 125mg PO BID (n=37) | Placebo (n=17) | None | 0/37 | 0/17 |
| Sildenafil | Iversen et al (2010)(54) | Randomized, double-blind, placebo-controlled crossover trial | 20 | 3 months | 50mg PO TID (n=10) | Placebo (n=10) | Bosentan (20) | 0/10 | 0/10 |
| Macitentan | MAESTRO (2018)(55) | Randomized, double-blind, placebo-controlled trial | 226 | 16 weeks | 10mg PO daily (n=114) | Placebo (n=112) | PDE5i (62) | 0/114 | 0/112 |
| **Portopulmonary hypertension** | | | | | | | | | |
| Macitentan | PORTICO (2019)(56) | Randomized, double-blind, placebo-controlled trial | 85 | 12 weeks | 10mg PO daily (n=43) | Placebo (n=42) | "Concurrent PAH therapy" (54) | 0/43 | 0/42 |

PDE5i = Phosphodiesterase type 5 inhibitor; PAH = pulmonary arterial hypertension

**Supplementary references**


1. Galiè N, Ghofrani HA, Torbicki A, Barst RJ, Rubin LJ, Badesch D, et al. Sildenafil citrate therapy for pulmonary arterial hypertension. N Engl J Med. 2005 Nov 17;353(20):2148–57.
2. Wilkins MR, Paul GA, Strange JW, Tunariu N, Gin-Sing W, Banya WA, et al. Sildenafil versus endothelin receptor antagonist for pulmonary hypertension (SERAPH) study. Am J Respir Crit Care Med. 2005 Jun;171(11):1292–7.
3. Singh TP, Rohit M, Grover A, Malhotra S, Vijayvergiya R. A randomized, placebo-controlled, double-blind, crossover study to evaluate the efficacy of oral sildenafil therapy in severe pulmonary artery hypertension. Am Heart J. 2006 Apr;151(4):851.e1–5.
4. Simonneau G, Rubin LJ, Galiè N, Barst RJ, Fleming TR, Frost AE, et al. Addition of sildenafil to long-term intravenous epoprostenol therapy in patients with pulmonary arterial hypertension. Ann Intern Med. 2008 Oct 21;149(8):521–30.
5. Vizza CD, Sastry BKS, Safdar Z, Harnisch L, Gao X, Zhang M, et al. Efficacy of 1, 5, and 20 mg oral sildenafil in adults with pulmonary arterial hypertension: a randomized, double-blind study with open-label extension. BMC Pulm Med. 2017 Feb;17(1):44.
6. Hoeper MM, Ewert R, Jansa P, Sirenko Y, Skride A, Balagtas C, et al. Randomized multicenter study to assess the effects of different doses of sildenafil on mortality in adults with pulmonary arterial hypertension. Circulation. 2024 Jun 18;149(25):1949–59.
7. Galiè N, Brundage BH, Ghofrani HA, Oudiz RJ, Simonneau G, Safdar Z, et al. Tadalafil therapy for pulmonary arterial hypertension. Circulation. 2009 Jun;119(22):2894–903.
8. Zhuang Y, Jiang B, Gao H, Zhao W. Randomized study of adding tadalafil to existing ambrisentan in pulmonary arterial hypertension. Hypertens Res. 2014 Jun;37(6):507–12.
9. Ghofrani HA, Galiè N, Grimminger F, Grünig E, Humbert M, Jing ZC, et al. Riociguat for the treatment of pulmonary arterial hypertension. N Engl J Med. 2013 Jul;369(4):330–40.
10. Rubin LJ, Galiè N, Grimminger F, Grünig E, Humbert M, Jing ZC, et al. Riociguat for the treatment of pulmonary arterial hypertension: a long-term extension study (PATENT-2). Eur Respir J. 2015 May;45(5):1303–13.
11. Galiè N, Müller K, Scalise AV, Grünig E. PATENT PLUS: a blinded randomized extension study of riociguat plus sildenafil in pulmonary arterial hypertension. Eur Respir J. 2015 May;45(5):1314–22.
12. Hoeper MM, Al-Hiti H, Benza RL, Chang SA, Corris PA, Gibbs JSR, et al. Switching to riociguat versus maintenance therapy with phosphodiesterase-5 inhibitors in pulmonary arterial hypertension (REPLACE). Lancet Respir Med. 2021 Jun;9(6):573–84.
13. Humbert M, Hassoun PM, Chin KM, Bortman G, Patel MJ, La Rosa C, et al. MK-5475, an inhaled soluble guanylate cyclase stimulator, for treatment of pulmonary arterial hypertension: the INSIGNIA-PAH study. Eur Respir J. 2024 Nov;64(5):2401110.
14. Galiè N, Olschewski H, Oudiz RJ, Torres F, Frost A, Ghofrani HA, et al. Ambrisentan for pulmonary arterial hypertension: results of the ARIES-1 and ARIES-2 studies. Circulation. 2008 Jun;117(23):3010–9.

15. Channick RN, Simonneau G, Sitbon O, Robbins IM, Frost A, Tapson VF, et al. Effects of the dual endothelin-receptor antagonist bosentan in pulmonary hypertension. Lancet. 2001;358(9288):1119–23.
16. Rubin LJ, Badesch DB, Barst RJ, Galiè N, Black CM, Keogh A, et al. Bosentan therapy for pulmonary arterial hypertension. N Engl J Med. 2002 Mar 21;346(12):896–903.
17. Galiè N, Rubin L, Hoeper M, Jansa P, Al-Hiti H, Meyer G, et al. Treatment of mildly symptomatic pulmonary arterial hypertension with bosentan (EARLY study). Lancet. 2008 Jun;371(9630):2093–100.
18. McLaughlin V, Channick RN, Ghofrani HA, Lemarié JC, Naeije R, Packer M, et al. Bosentan added to sildenafil therapy in pulmonary arterial hypertension. Eur Respir J. 2015 Aug;46(2):405–13.
19. Pulido T, Adzerikho I, Channick RN, Delcroix M, Galiè N, Ghofrani HA, et al. Macitentan and morbidity and mortality in pulmonary arterial hypertension. N Engl J Med. 2013 Aug;369(9):809–18.
20. Barst RJ, Langleben D, Frost A, Horn EM, Oudiz R, Shapiro S, et al. Sitaxsentan therapy for pulmonary arterial hypertension. Am J Respir Crit Care Med. 2004 Feb;169(4):441–7.
21. Barst RJ, Langleben D, Badesch D, Frost A, Lawrence EC, Shapiro S, et al. Treatment of pulmonary arterial hypertension with the selective endothelin-A receptor antagonist sitaxsentan. J Am Coll Cardiol. 2006 May;47(10):2049–56.
22. Simonneau G, Torbicki A, Hoeper MM, Delcroix M, Karlócai K, Galiè N, et al. Selexipag: an oral selective prostacyclin receptor agonist for pulmonary arterial hypertension. Eur Respir J. 2012 Oct;40(4):874–80.
23. Sitbon O, Channick R, Chin KM, Frey A, Gaine S, Galiè N, et al. Selexipag for the treatment of pulmonary arterial hypertension. N Engl J Med. 2015 Dec;373(26):2522–33.
24. Howard LS, Rosenkranz S, Frantz RP, Hemnes AR, Pfister T, Hsu Schmitz SF, et al. Assessing daily life physical activity by actigraphy in pulmonary arterial hypertension (TRACE). Chest. 2023 Feb;163(2):407–18.
25. Torres F, Farber H, Ristic A, McLaughlin V, Adams J, Zhang J, et al. Efficacy and safety of ralinepag in pulmonary arterial hypertension: a phase 2 randomized trial. Eur Respir J. 2019 Jan;53:1901030.
26. Barberà J, Jansa P, Klings E, Ristić A, Keogh A, Solum D, et al. Ralinepag phase II open-label extension study in pulmonary arterial hypertension. Adv Ther. 2024 Mar;41(3):1062–74.
27. Barst RJ, Rubin LJ, Long WA, McGoon MD, Rich S, Badesch DB, et al. Comparison of continuous intravenous epoprostenol with conventional therapy for primary pulmonary hypertension. N Engl J Med. 1996 Feb 1;334(5):296–301.
28. Tapson VF, Torres F, Kermeen F, Keogh AM, Allen RP, Frantz RP, et al. Oral treprostinil for pulmonary arterial hypertension on background therapy (FREEDOM-C). Chest. 2012 Dec;142(6):1383–90.
29. Tapson VF, Jing ZC, Xu KF, Pan L, Feldman J, Kiely DG, et al. Oral treprostinil for pulmonary arterial hypertension on background therapy (FREEDOM-C2). Chest. 2013;144(3):952–8.

30. Jing ZC, Parikh K, Pulido T, Jerjes-Sanchez C, White RJ, Allen R, et al. Oral treprostinil monotherapy for pulmonary arterial hypertension. Circulation. 2013 Feb 5;127(5):624–33.
31. White RJ, Jerjes-Sanchez C, Bohns Meyer GM, Pulido T, Sepulveda P, Wang KY, et al. Combination therapy with oral treprostinil for pulmonary arterial hypertension. Am J Respir Crit Care Med. 2020 Mar;201(6):707–17.
32. Simonneau G, Barst RJ, Galiè N, Naeije R, Rich S, Bourge RC, et al. Continuous subcutaneous treprostinil infusion in pulmonary arterial hypertension. Am J Respir Crit Care Med. 2002 Mar;165(6):800–4.
33. McLaughlin VV, Benza RL, Rubin LJ, Channick RN, Voswinckel R, Tapson VF, et al. Addition of inhaled treprostinil to oral therapy in pulmonary arterial hypertension. J Am Coll Cardiol. 2010 May;55(18):1915–22.
34. Olschewski H, Simonneau G, Galiè N, Higenbottam T, Naeije R, Rubin LJ, et al. Inhaled iloprost for severe pulmonary hypertension. N Engl J Med. 2002 Aug;347(5):322–9.
35. McLaughlin VV, Oudiz RJ, Frost A, Tapson VF, Murali S, Channick RN, et al. Adding inhaled iloprost to bosentan in pulmonary arterial hypertension. Am J Respir Crit Care Med. 2006 Dec;174(11):1257–63.
36. Galiè N, Humbert M, Vachiéry JL, Vizza CD, Kneussl M, Manes A, et al. Beraprost sodium in pulmonary arterial hypertension: a randomized trial. J Am Coll Cardiol. 2002 May;39(9):1496–502.
37. Barst RJ, McGoon M, McLaughlin V, Tapson V, Rich S, Rubin L, et al. Beraprost therapy for pulmonary arterial hypertension. J Am Coll Cardiol. 2003 Jun;41(12):2119–25.
38. Humbert M, McLaughlin V, Gibbs JSR, Gomberg-Maitland M, Hoeper MM, Preston IR, et al. Sotatercept for pulmonary arterial hypertension. N Engl J Med. 2021 Apr;384(13):1204–15.
39. Humbert M, McLaughlin V, Gibbs JSR, Gomberg-Maitland M, Hoeper MM, Preston IR, et al. Sotatercept for pulmonary arterial hypertension: PULSAR open-label extension. Eur Respir J. 2023 Jan;61(1).
40. Hoeper MM, Badesch DB, Ghofrani HA, Gibbs JSR, Gomberg-Maitland M, McLaughlin VV, et al. Phase 3 trial of sotatercept in pulmonary arterial hypertension. N Engl J Med. 2023 Apr 20;388(16):1478–90.
41. Humbert M, McLaughlin VV, Badesch DB, Ghofrani HA, Gibbs JSR, Gomberg-Maitland M, et al. Sotatercept in high-risk pulmonary arterial hypertension. N Engl J Med. 2025 May 29;392(20):1987–2000.
42. McLaughlin VV, Hoeper MM, Badesch DB, Ghofrani HA, Gibbs JSR, Gomberg-Maitland M, et al. Sotatercept within the first year after pulmonary arterial hypertension diagnosis. N Engl J Med. 2025 Oct 23;393(16):1599–611.
43. Ghofrani HA, Morrell NW, Hoeper MM, Olschewski H, Peacock AJ, Barst RJ, et al. Imatinib in pulmonary arterial hypertension with inadequate treatment response. Am J Respir Crit Care Med. 2010 Nov;182(9):1171–7.
44. Hoeper MM, Barst RJ, Bourge RC, Feldman J, Frost AE, Galiè N, et al. Imatinib mesylate add-on therapy in pulmonary arterial hypertension (IMPRES). Circulation. 2013 Mar;127(10):1128–38.

45. Hill NS, Gillies H, Chakinala MM, Feldman JP, Hoeper MM, Jing ZC, McLaughlin VV, Rosenkranz S, Escribano-Subias P, Ghio S, Gomberg-Maitland M, Habib N, Kopec G, Langleben D, Lescano A, Ramirez A, Rischard F, Sitbon O, Skride A, Dake BT, Khindri S, Langley J, Niven R, Zhang X, Humbert M. Phase 2b trial of inhaled imatinib for treatment of pulmonary arterial hypertension. Am J Respir Crit Care Med 2026; Jan 23. In press.
46. Frantz RP, McLaughlin VV, Sahay S, Escribano Subías P, Zolty RL, Benza RL, et al. Seralutinib in pulmonary arterial hypertension (TORREY). Lancet Respir Med. 2024 Jul;12(7):523–34.
47. Humbert M, Barst RJ, Robbins IM, Channick RN, Galiè N, Boonstra A, et al. Bosentan combined with epoprostenol in pulmonary arterial hypertension (BREATHE-2). Eur Respir J. 2004 Sep;24(3):353–9.
48. Hoeper MM, Leuchte H, Halank M, Wilkens H, Meyer FJ, Seyfarth HJ, et al. Inhaled iloprost plus bosentan in idiopathic pulmonary arterial hypertension. Eur Respir J. 2006 Oct;28(4):691–4.
49. Galiè N, Barberà JA, Frost AE, Ghofrani HA, Hoeper MM, McLaughlin VV, et al. Initial ambrisentan plus tadalafil therapy in pulmonary arterial hypertension. N Engl J Med. 2015 Aug 27;373(9):834–44.
50. Chin KM, Sitbon O, Doelberg M, Feldman J, Gibbs JSR, Grünig E, et al. Three- versus two-drug therapy for newly diagnosed pulmonary arterial hypertension. J Am Coll Cardiol. 2021 Oct;78(14):1393–403.
51. Ghofrani HA, Jansa P, Frost A, Hoeper MM, Humbert M, McLaughlin VV, et al. Macitentan/tadalafil single-tablet combination therapy for pulmonary arterial hypertension. J Am Coll Cardiol. 2024 Jan 30;83(4):473–84.
52. Badesch DB, Tapson VF, McGoon MD, Brundage BH, Rubin LJ, Wigley FM, et al. Continuous intravenous epoprostenol in scleroderma-associated pulmonary hypertension. Ann Intern Med. 2000 Mar;132(6):425–34.
53. Galiè N, Beghetti M, Gatzoulis MA, Granton J, Berger RMF, Lauer A, et al. Bosentan therapy in Eisenmenger syndrome. Circulation. 2006 Jul;114(1):48–54.
54. Iversen K, Jensen AS, Jensen TV, Vejlstrup NG, Søndergaard L. Bosentan and sildenafil combination therapy in Eisenmenger syndrome. Eur Heart J. 2010 May;31(9):1124–31.
55. Gatzoulis MA, Landzberg M, Beghetti M, Berger RM, Efficace M, Gesang S, et al. Macitentan in Eisenmenger syndrome. Circulation. 2019 Jan 2;139(1):51–63.
56. Sitbon O, Bosch J, Cottreel E, Csonka D, de Groote P, Hoeper MM, et al. Macitentan for portopulmonary hypertension (PORTICO). Lancet Respir Med. 2019 Jul;7(7):594–604.
